# Hopf Bifurcations of Moore-Greitzer PDE Model with Additive Noise

**DOI:** 10.1007/s00332-023-09929-7

**Published:** 2023-06-17

**Authors:** Yiming Meng, N. Sri Namachchivaya, Nicolas Perkowski

**Affiliations:** 1 Department of Applied Mathematics, Waterloo University, Waterloo, ON Canada; 2 Institut für Mathematik, Freie Universität Berlin, Berlin, DE Germany

**Keywords:** Moore-Greitzer PDE model, Additive noise, Hopf bifurcation, Stall, Multiscale analysis, Low-dimensional approximations, 60H30

## Abstract

The Moore-Greitzer partial differential equation (PDE) is a commonly used mathematical model for capturing flow and pressure changes in axial-flow jet engine compressors. Determined by compressor geometry, the deterministic model is characterized by three types of Hopf bifurcations as the throttle coefficient decreases, namely surge (mean flow oscillations), stall (inlet flow disturbances) or a combination of both. Instabilities place fundamental limits on jet-engine operating range and thus limit the design space. In contrast to the deterministic PDEs, the Hopf bifurcation in stochastic PDEs is not well understood. The goal of this particular work is to rigorously develop low-dimensional approximations using a multiscale analysis approach near the deterministic stall bifurcation points in the presence of additive noise acting on the fast modes. We also show that the reduced-dimensional approximations (SDEs) contain multiplicative noise. Instability margins in the presence of uncertainties can be thus approximated, which will eventually lead to lighter and more efficient jet engine design.

## Introduction

Jet engine compressors can exhibit instabilities near their optimal operating range, which reduce performance and are potentially dangerous. One of these instabilities is rotating stall, whereby the circumferential flow pattern is disturbed. This manifests itself as a region of severely reduced flow that rotates at a fraction of the rotor speed and causes a drop in performance. A second instability is surge, a pumping oscillation that can cause flame-out and engine damage. The detection of compressor instabilities (surge, stall or a combination of the two) is essential for increasing compressor efficiency, preventing damage or even failure, and lengthening the overall life-span of the engine components.

Moore and Greitzer (1986), Greitzer and Moore (1986) developed a relatively simple set of equations that model airflow through the compression system of a jet engine. This mathematical model consists of a PDE that describes the behavior of disturbances in the inlet region of compression systems, and two ODEs that describe the coupling of the disturbances within the mean flow. The model is also equipped with boundary conditions to express the pressure rise between the upstream reservoir and the exit duct discharge. Furthermore, Birnir et al. (2007) used the stochastic homogenization theory of fluids to derive a modified version from the Navier–Stokes equations.

The deterministic Moore-Greitzer PDE model can be converted in general into an abstract evolution parabolic PDE (Banaszuk et al. 1999),

$$\begin{aligned} \partial _{t}u(t) =Au(t)+f(\mu ,u(t)),\;\;\;u(0)=u_0, \end{aligned}$$

where for every $t\in [0,\infty )$, $u(t)$ takes value in a product Hilbert space $U:={\mathcal {H}}\times {\mathbb {R}}\times {\mathbb {R}}$ with ${\mathcal {H}}$ as an infinite-dimensional separable Hilbert space. The unbounded linear operator *A* equipped with certain boundary conditions generates an analytic compact $C_0$ semigroup on *U*. The field $f(\mu ,u)$ contains cubic polynomials that also depend on the parameter $\mu$. Linearization around an equilibrium point $u_e(\mu )$ gives rise to the linear operator $A+Df_{u_e}(\mu )$. It has been verified that $A+Df_{u_e}(\mu )$ only admits a point spectrum, i.e., $\sigma (A+Df_{u_e}(\mu ))=\sigma _p(A+Df_{u_e}(\mu ))=\{\rho _{\pm k},\;\forall k\in {\mathcal {I}}\}$, for a certain index set ${\mathcal {I}}$ (Xiao and Basar 2000). That the eigenvalues $\rho _{\pm k}$ appear in conjugate pairs is attributed to the spiral structure of the phase flow.

To make the analysis less cumbersome, we work on the localized model with topological equivalence

1.1 $$\begin{aligned} \partial _t v=(A+Df_{u_e})(\mu )v+B(v,v)+{{\textbf {F}}}(v,v,v), \end{aligned}$$

where, for each $\mu$, $v=u-u_e(\mu )$ is the perturbation around $u_e(\mu )$, the operators $B(\cdot ,\cdot )$ and ${{\textbf {F}}}(\cdot ,\cdot ,\cdot )$ represent respectively bilinear and trilinear mappings. As for the system (1.1), the new equilibrium point is always $\textbf{0}$ (the trivial fixed point) for all $\mu \in \mathbb {R}$. The system exhibits three types of Hopf bifurcations (Xiao 2008), that is, at some critical $\mu _c$, we have ${\text {Re}}[\rho _{\pm k}({A+Df_{u_e}(\mu _c))}]=0$ but $\left. \frac{d{\text {Re}}[\rho _{\pm k}({A+Df_{u_e}(\mu ))}]}{d\mu }\right| _{\mu =\mu _c}\ne 0$ for the associated critical $k\in {\mathcal {I}}_c\subset {\mathcal {I}}$, while the rest of the spectrum stays in the left half-plane. In the above setting, we are particularly interested in the local behaviour of the system near $\textbf{0}$, parametrized by $\mu$ in some small neighborhood of $\mu _c$. The local stability of the hyperbolic equilibrium points $v_e(\mu )$, is determined by the sign of the real part of the eigenvalues of $A+Df_{u_e}(\mu )$. However, at a bifurcation point $\mu _c$, the linear operator $A+Df_{u_e}(\mu _c)$ does not provide any information about exponential convergence (or divergence) of the system. The slowly-varying dynamics on the center manifold must be investigated to study the nonlinear effects on determining the stability of the system.

To simplify the notation, the eigenvalues of $(A+Df_{u_e}(\mu ))|_{\mathcal {H}}$ will be denoted by $\lambda _{\pm k}(\mu )$, with ${\text {Re}}(\lambda _{\pm k})$ decreasing in *k*, and those of $(A+Df_{u_e}(\mu ))|_{\mathbb {R}^2}$ by $\gamma _{\pm 1}(\mu )$. The corresponding eigenvalues $\lambda _{\pm 1}$ and $\gamma _{\pm 1}$ will pass through a change of stability independently. Depending on which pair of eigenvalues crosses the imaginary axis first as the bifurcation parameter $\mu$ varies, there are three possible types of Hopf bifurcations: If $\lambda _{\pm 1}$ crosses the imaginary axis first, the physical oscillations are dominated by stall effects; if $\gamma _{\pm 1}$ satisfies the Hopf bifurcation condition, then surge effects dominate; if $\lambda _{\pm 1}$ and $\gamma _{\pm 1}$ cross the imaginary axis simultaneously, we see a mixture of both effects. Xiao (2008) has verified that the oscillation type is only determined by the fluid’s viscosity and the geometric structure of the compressors.

The existence of the center manifold for the deterministic Moore-Greitzer model is well understood (Xiao and Basar 2000). The evolution of states on the center manifold is studied by naturally separating the dynamics into critical modes and fast modes. The critical subspaces are given as $U^{\text {stall}}_c=[{\text {span}}\{e^{\pm i\theta }\},0,0]^T$, $U^\text {surge}_c=\mathbb {R}^2$, and $U^\text {mix}_c=U^\text {stall}_c\oplus U^\text {surge}_c$, respectively, where the subscript *c* denotes ‘critical’, and the superscripts describe the types of engine instabilities. If we denote the orthogonal projection by $P_c: U\rightarrow U^\text {stall}_c$ (resp. $P_c: U\rightarrow U^\text {surge}_c$ or $P_c: U\rightarrow U^\text {mix}_c$), as well as $P_s:=I-P_c$, the solution can be represented as $U\ni v=x+y$ with $x\in P_cU$ and $y\in P_sU$. Therefore, (1.1) can be converted into an equivalent form:

1.2 $$\begin{aligned} \begin{aligned}&\partial _{t} x=P_c(A+Df_{u_e})(\mu )v+P_c[B(v,v)+{{\textbf {F}}}(v,v,v)];\\&\partial _{t} y=P_s(A+Df_{u_e})(\mu )v+P_s[B(v,v)+{{\textbf {F}}}(v,v,v)]. \end{aligned} \end{aligned}$$

Note that the term $P_c(A+Df_{u_e})(\mu )$ depends linearly on $\mu$, while even for $\mu =\mu _c$ the term $P_s(A+Df_{u_e})(\mu _c)v$ does not vanish. This suggests that in the neighborhood of $\mu _c$ the function *y* evolves much faster than *x*. The analytical center manifold determines the long-time behavior of *y* as a smooth mapping *h* of *x* (Guckenheimer and Holmes 2013), i.e. $\lim \limits _{t\rightarrow \infty }y(t)=h(x)$. Therefore, the dominating dynamics restricted to $P_cU$ depends only on *x*:

1.3 $$\begin{aligned} \partial _{t} x= & {} P_c(A+Df_{u_e})(\mu )v+P_c[B(x+h(x),x+h(x))\nonumber \\{} & {} +{{\textbf {F}}}(x+h(x),x+h(x),x+h(x))]. \end{aligned}$$

In addition, $z_j=\langle \zeta _j, x\rangle \in {\mathbb {C}}$ for all eigenvector $\zeta _j\in P_cU$, solve a ${\text {dim}}(P_cU)$-dimensional amplitude equation that is equivalent to (1.3).

In contrast to the deterministic model, the Hopf bifurcation in stochastic partial differential equations (SPDEs) is not well understood (Arnold et al. 1996; Baxendale 1994). Given an appropriate probability space $(\varOmega , {\mathscr {F}}, {\mathbb {P}})$, the evolution of the axial flow in an engine compressor with unsteady turbulence is modelled by the abstract Moore-Greitzer stochastic PDE, written locally as

1.4 $$\begin{aligned} dv =(A+Df_{u_e})(\mu )vdt+vB(v,v)dt+\text {F}(v,v,v)dt+\varepsilon dW_{t},\;\;\;v(0)=v_0, \nonumber \\ \end{aligned}$$

where $\dot{W_{t}}(\omega )$ represents the effect of turbulence (Kim and Abed 1999; Gourdain et al. 2014), modeled by an additive Gaussian noise (white in time, either white or colored in space) with a small strength $\varepsilon$. The random perturbations are small, but over a long time their effect can be significant on the slow dynamics of the amplitudes of the critical modes. It is worth remarking that instead of using finite-dimensional noise that only acts on one of the stable modes as in Blömker and Romito (2015), we use the infinite-dimension Gaussian-type noise (see examples in Def. 14) with appropriate space-time regularity conditions. Such a modelling setup has a reasonable physical meaning, and is also amenable for the analysis and derivations presented in Sect. 5.

In this paper, a two-dimensional SDE, regarded as the stochastic amplitude equations of the dominant dynamics, are derived for the stall bifurcation. We achieve this by investigating ${\hat{v}}(t):=\varepsilon ^{-1}v(\varepsilon ^{-2}t)$ that solves

1.5 $$\begin{aligned} \boxed {d{\hat{v}} =\varepsilon ^{-2}(A+Df_{u_e})(\mu ){\hat{v}}dt+\varepsilon ^{-1}B({\hat{v}},{\hat{v}})dt+\text {F}({\hat{v}},{\hat{v}},{\hat{v}})dt+\varepsilon ^{-1} d{\hat{W}}_{t},\;\;\;{\hat{v}}(0)={\hat{v}}_0,} \end{aligned}$$

where ${\hat{W}}_t:=\varepsilon W_{\varepsilon ^{-2}t}$ is a new Wiener process, and ${\mu =\mu _c+\varepsilon ^2\mathfrak {q}}$ for some $\mathfrak {q}\in \mathbb {R}$. Due to the natural separation of the temporal scales close to the deterministic bifurcation points, the work is based on a multiscale analysis of the coupling between the slow and fast modes as an extension of Blömker et al. (2007). Our goal in this paper is to extend the work of Blömker et al. (2007) and Blömker and Hongbo (2020) and develop multiscale methods to study the effects of turbulence on the flow oscillations. The derivation is provided explicitly for the purpose of engineering applications. We expect the results will motivate engineers with theoretical background and shed some light on the design of lighter and more efficient jet engines.

Denoting the solution of (1.5) by ${\hat{v}}(t)=[\hat{g}(t),{\hat{\varPhi }}_\delta (t),{\hat{\varPsi }}_\delta (t)]^T\in U$, we focus our attention on the systems where the parameters are in the vicinity of stall bifurcation point $\mu _c$. The main result of the paper is the following:

### Theorem 1

Under the assumptions stated in Sect. 3, given $\mu =\mu _c+\varepsilon ^2\mathfrak {q}$ for some parameter $\mathfrak {q}$, an approximation of the slowly-varying dynamics of $\hat{g}(t)\in {\mathcal {H}}$ at ${\hat{\mu }}$ is obtained by

$$\begin{aligned} P_c\hat{g}(t)=\hat{z}(t)e^{i\theta }+\overline{\hat{z}}(t)e^{-i\theta }, \end{aligned}$$

where $v^a:= [Re(\hat{z}),Im(\hat{z})]^T$ solves a two-dimensional SDE of the form

1.6 $$\begin{aligned} \begin{aligned}&v^a(t)=v^a(0)+\int _0^t{{\mathfrak {A}}(\mathfrak {q})}v^a(s)ds+\int _0^t|v^a(s)|^2{\mathfrak {B}}v^a(s)ds+\int _0^tM(v^a(s))d{\mathcal {W}}_s{+{\text {Er}}(t)};\\&v^a(0)=[Re(\hat{z}(0)),Im(\hat{z}(0)]^T;\\&{{\mathbb {E}}\left[ \sup _{t\in [0,\tau ^*]} \Vert {\text {Er}}(t)\Vert ^p\right] ={\mathcal {O}}(\varepsilon ^{p/2-})}, \end{aligned} \end{aligned}$$

where the matrices ${\mathfrak {A}}(\mathfrak {q}),{\mathfrak {B}}$ and *M* as well as the driving force ${\mathcal {W}}$ are defined in Sect. 5. The stochastic effects appear multiplicatively in the last term above. The quantity $\tau ^*$ is a stopping time up to some $T>0$ such that, given negative diagonal entries of ${\mathfrak {B}}$, ${\mathbb {P}}[\tau ^*\le t]\rightarrow 0$ as $\varepsilon \rightarrow 0$ for all $t\in (0,T]$.

Let $\nu _c^{\varepsilon }$ be the law of $\{v^a(t\wedge \tau ^*)\}_{t\le T}$. Then, as $\varepsilon \rightarrow 0$, the sequence of $\nu _c^{\varepsilon }$ converges weakly to the measure $\nu _c$, which is the law of the solution to

1.7 $$\begin{aligned} {\tilde{v}}^a={\tilde{v}}^a(0)+\int _0^{t}{\mathfrak {A}}(\mathfrak {q}){\tilde{v}}^ads+\int _0^{t}|{\tilde{v}}^a|^2{\mathfrak {B}}{\tilde{v}}^ads+\int _0^{t}\varSigma ({\tilde{v}}^a)d\beta _s, \end{aligned}$$

where $\beta _t$ is a two-dimensional Brownian motion, and $\varSigma$ is defined in (6.2).

### Remark 2

To succinctly convey the methodology, we shall hereby only consider the stall case of the three possible instabilities in the Moore-Greitzer model. The approximation for the surge and stall-surge cases can be done by the same method, but with different rescaling schemes. A short discussion is provided in Remark 23.

The rest of the paper is organized as follows. In Sect. 2, we formally review the physical model and recast it into the form of (1.3) for the stall case. The discussion of the stochastic model is based on this. In Sect. 3, the assumptions for the stochastic analysis will be stated. We describe the behavior of the stochastic Moore-Greitzer PDE model with the setup stall parameters before state explosion, derive the finite-dimensional approximation, prove the error bound, and show the weak convergence result from Sect. 4 to Sect. 6. The conclusions follow in Sect. 7.

## Deterministic Moore-Greitzer Model

**Fig. 1:**
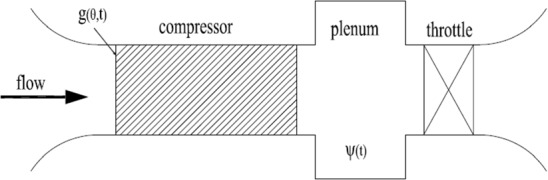
Compression system

**Fig. 2:**
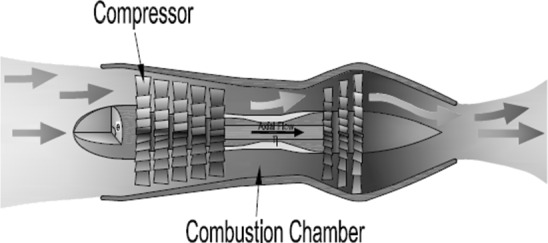
Compressor geometry

The structure of the compression system and the compressor geometry are given in Figs. 1 and 2.

The compressor gives pressure rise to the upstream flow and sends it into the plenum through the downstream duct. The throttle controls the averaged mass flow through the system at the rear of the plenum. The stability of the compression system is twofold: (stall) the upstream non-uniform disturbance generates a locally higher angle of attack, and propagates along the blade row without mitigation; (surge) the average mean flow and pressure rise oscillate constantly and formulate standing waves (Gravdahl 1998). The deterministic Moore-Greitzer model captures the dynamic evolution of the above states, and is given explicitly as Xiao (2008):

2.1 $$\begin{aligned} \frac{\partial }{\partial t}\begin{bmatrix} g\\ \varPhi \\ \varPsi \end{bmatrix}=\begin{bmatrix} K^{-1}(\frac{\nu }{2}\frac{\partial ^2}{\partial \theta ^2}-\frac{1}{2}\frac{\partial }{\partial \theta }) &{} 0 &{} 0\\ 0 &{} 0 &{} 0\\ 0 &{} 0 &{} 0\\ \end{bmatrix}\begin{bmatrix} g\\ \varPhi \\ \varPsi \end{bmatrix} +\begin{bmatrix} aK^{-1}(\psi _c(\varPhi +g)-{\overline{\psi }}_c{(\varPhi ,g)})\\ \frac{1}{l_c}({\overline{\psi }}_c{(\varPhi ,g)}-\varPsi )\\ \frac{1}{4l_cB^2}(\varPhi -\mu \sqrt{\varPsi }) \end{bmatrix}, \end{aligned}$$

where the states $[g(t),\varPhi (t),\varPsi (t)]^T\in U:={\mathcal {H}}\times {\mathbb {R}}\times {\mathbb {R}}$ are as introduced before. The physical meaning of the states are as follows, $g(t,\theta )$ represents the velocity of upstream disturbance along the axial direction at the duct entrance, $\varPhi (t)$ is the averaged mean flow rate, $\varPsi (t)$ is the averaged pressure. We require that $g(t,0)=g(t,2\pi )$, $g_{\theta }(t,0)=g_{\theta }(t,2\pi )$ and $\int _0^{2\pi } g(\tau ,\theta )d\theta =0$, thus,

$$\begin{aligned} g(t,\theta )=\sum \limits _{n\in {\mathbb {Z}}\setminus \{0\}}g_n(t)e^{in\theta }. \end{aligned}$$

The operator *K* is defined as a Fourier multiplier,

$$\begin{aligned} K(g)=\sum \limits _{n\in {\mathbb {Z}}\setminus \{0\}}\left\{ 1+\frac{am}{|n|}\right\} g_n(t)e^{in\theta }, \end{aligned}$$

where *a* is the internal compressor lag and *m* is the duct parameter. The compressor characteristic $\psi _c$ is given in a cubic form,

2.2 $$\begin{aligned} \psi _c(\varPhi )=\psi _{c_0}+\iota \left[ 1+\frac{3}{2}\left( \frac{\varPhi }{\varTheta }-1\right) -\frac{1}{2}\left( \frac{\varPhi }{\varTheta }-1\right) ^3 \right] \end{aligned}$$

where $\psi _{c_0}$, $\iota$ and $\varTheta$ are real-valued parameters that are defined by the compressor configuration. We also define

$$\begin{aligned} {\overline{\psi }}_c{(\varPhi ,g)}: =\frac{1}{2\pi }\int _0^{2\pi }\psi _c(\varPhi +g)d\theta . \end{aligned}$$

As for the other parameters, $l_c>0$ is the compressor length, $B>0$ is the plenum-to-compressor volume ratio, $\nu >0$ is the viscous coefficient. The parameter $\mu$ represents the throttle coefficient, the decrease of which will cause the stability change.

### Remark 3

The solution of $g(t)$ lies in an infinite-dimensional Hilbert space ${\mathcal {H}}:=\{h \in L^2[0,2\pi ]: \int _0^{2\pi } h(\theta ) d\theta = 0\}$ equipped with the inner product

2.3 $$\begin{aligned} \langle h_1,h_2\rangle _{{\mathcal {H}}}:=\langle h_1, Kh_2\rangle , \;h_1,h_2\in \mathcal {H}, \end{aligned}$$

as well as the induced norm $\Vert \cdot \Vert _{{\mathcal {H}}}$; note that the Fourier multiplier ${K:\mathcal {H}\rightarrow \mathcal {H}}$ is a positive definite and self-adjoint linear operator. More details on the operator *K* and $K^{-1}$ can be found in Xiao (2008). We also identify $\mathcal {H}$ with its dual through the Riesz isomorphism. In general, due to the spatial periodicity and the zero-average property ($\int _0^{2\pi }g(t,\theta )d\theta =0$ for all *t*), we can expect the solution *g*(*t*) to be at least in a Sobolev space $H^2_{0}\subset {\mathcal {H}}$, which is formally defined Def. 9. The space $U = {\mathcal {H}}\times {\mathbb {R}} \times {\mathbb {R}}$ is then a product Hilbert space with inner product defined by

2.4 $$\begin{aligned} \langle u_1,u_2\rangle _{U} = \langle (g_1,\varPhi _1,\varPsi _1),(g_2,\varPhi _2,\varPsi _2)\rangle _{U}\!:=\!\langle g_1,g_2\rangle _{{\mathcal {H}}}\!+\!l_c\varPhi _1\varPhi _2+(4l_cB^2)\varPsi _1\varPsi _2.\nonumber \\ \end{aligned}$$

### Abstract Form

In abstract form we can write (2.1) as

2.5 $$\begin{aligned} \partial _{t}u=Au+f(\mu ,u), \end{aligned}$$

where $u=[g,\varPhi ,\varPsi ]^T\in U$, *A* is the operator matrix

$$\begin{aligned} A=\begin{bmatrix} K^{-1}\left( \frac{\nu }{2}\frac{\partial ^2}{\partial \theta ^2}-\frac{1}{2}\frac{\partial }{\partial \theta }\right) &{} 0 &{} 0\\ 0 &{} 0 &{} 0\\ 0 &{} 0 &{} 0\\ \end{bmatrix}, \end{aligned}$$

and

$$\begin{aligned} f(\mu ,u)=\begin{bmatrix} aK^{-1}(\psi _c(\varPhi +g)-{\overline{\psi }}_c{(\varPhi ,g)})\\ \frac{1}{l_c}({\overline{\psi }}_c{(\varPhi ,g)}-\varPsi )\\ \frac{1}{4l_cB^2}(\varPhi -\mu \sqrt{\varPsi }) \end{bmatrix}. \end{aligned}$$

We consider a fixed point of the form $u_e(\mu )=[0,\varPhi _e(\mu ),\varPsi _e(\mu )]^T$ and such that $f(\mu ,u_e(\mu ))={\textbf{0}}$ for each $\mu$. In particular $(\varPhi _e(\mu ),\varPsi _e(\mu ))$ is determined by the intersection of the compressor characteristic $\varPsi =\psi _c(\varPhi )$ and the throttle characteristic $\varPhi =\mu \sqrt{\varPsi }$.

#### Remark 4

Note that by definition, we have the following expansion:

$$\begin{aligned}{} & {} \psi _c(\varPhi +g)=\psi _c(\varPhi )\nonumber \\{} & {} \quad +\iota \left[ \frac{3}{2}\left( \frac{g}{\varTheta }\right) -\frac{1}{2}\left( \frac{g}{\varTheta }\right) ^3-\frac{3}{2}\left( \frac{\varPhi }{\varTheta }-1\right) ^2\frac{g}{\varTheta }-\frac{3}{2} \left( \frac{\varPhi }{\varTheta }-1\right) \left( \frac{g}{\varTheta }\right) ^2 \right] \end{aligned}$$

Since $$g=\sum _{n\in {\mathbb {Z}}\setminus \{0\}} g_n e^{in\theta }$$,

$$\begin{aligned} \begin{aligned} {\bar{\psi }}_c{(\varPhi ,g)}&=\frac{1}{2\pi }\int _0^{2\pi }\psi _c(\varPhi +g)d\theta =\frac{1}{2\pi }\int _0^{2\pi }\psi _c(\varPhi )d\theta \\&\quad +\frac{1}{2\pi }\int _0^{2\pi }\iota \left[ \frac{3}{2}\left( \frac{g}{\varTheta }\right) -\frac{1}{2}\left( \frac{g}{\varTheta }\right) ^3-\frac{3}{2}\left( \frac{\varPhi }{\varTheta } -1\right) ^2\frac{g}{\varTheta }-\frac{3}{2} \left( \frac{\varPhi }{\varTheta }-1\right) \left( \frac{g}{\varTheta }\right) ^2 \right] d\theta \\&= \psi _c(\varPhi ) -\frac{3\iota }{2\varTheta ^2}\left( \frac{\varPhi }{\varTheta }-1\right) \mathop {\sum }\limits _{\begin{array}{c} j,k\in {\mathbb {Z}}_0 \\ k+j=0 \end{array} }g_jg_k-\frac{\iota }{6\varTheta ^3}\mathop {\sum }\limits _{\begin{array}{c} j,k,l\in {\mathbb {Z}}_0 \\ k+j+l=0 \end{array} }g_jg_kg_l\\&=\psi _c(\varPhi ) +\frac{\psi _c''(\varPhi )}{2}\varPi ^{(2)}g^2+\frac{\psi _c'''(\varPhi )}{6}\varPi ^{(3)}g^3, \end{aligned} \end{aligned}$$

where we have used notations $$\varPi ^{(2)}uv=\mathop {\sum }\limits _{\begin{array}{c} j,k\in {\mathbb {Z}}_0 \\ k+j=0 \end{array} }u_jv_k$$ and $$\varPi ^{(3)}uvw=\mathop {\sum }\limits _{\begin{array}{c} j,k,l\in {\mathbb {Z}}_0 \\ k+j+l=0 \end{array} }u_jv_kw_l$$ for all $u,v,w\in H^2_{{\text {per}}}$. Therefore, the noisy perturbation of *g* that will be added in the next section enters the flow equations via $\varPi ^{(2)}g^2$ and $\varPi ^{(3)}g^3$. However, the operation points of the compressor, a family of stable fixed points $(\varPhi _e(\mu ),\varPsi _e(\mu ))$, are not influenced by *g*.

For local analysis in the neighborhood of $\mu _c$ (a bifurcation point of the original system), given a specified parameter $\mu$ , we define the unfolding parameter in this abstract setting as

$$\begin{aligned} {{\hat{\mathfrak {q}}}}:=\mu -\mu _c, \end{aligned}$$

which measures the distance from the true bifurcation point in the parameter space. We transform (2.5) into a topologically equivalent system by expanding $f(\mu , u_e(\mu ))$ locally w.r.t. each $u_e(\mu )$ up to $3^{\text {rd}}$-order terms, which results in the equation

2.6 $$\begin{aligned} \partial _t v=L({{\hat{\mathfrak {q}}}})v+B(v,v)+{{\textbf {F}}}(v,v,v), \end{aligned}$$

where $v=u-u_e(\mu )=[g,\varPhi _\delta ,\varPsi _\delta ]$ is the perturbation around $u_e(\mu )$, and $L({{\hat{\mathfrak {q}}}})$ is the linear operator given as

$$\begin{aligned} L({{\hat{\mathfrak {q}}}}):=A+Df_{u_e}({\mu _c+{\hat{\mathfrak {q}}}}). \end{aligned}$$

The Fréchet derivative at $u_e(\mu )$ is

2.7 $$\begin{aligned} Df_{u_e}({\mu _c+{\hat{\mathfrak {q}}}})=\begin{bmatrix} a(\psi _{c,\mu _c}'+\psi _{c,\mu _c}''\varPhi _{e,c}'{{\hat{\mathfrak {q}}}})K^{-1} &{} 0 &{} 0\\ 0 &{} \frac{1}{l_c}(\psi _{c,\mu _c}'+\psi _{c,\mu _c}''\varPhi _{e,c}'{{\hat{\mathfrak {q}}}}) &{} -\frac{1}{l_c}\\ 0 &{} \frac{1}{4B^2l_c} &{} \frac{1}{4B^2l_c}(\mathcal {S}_{\mu _c}'+\mathcal {S}_{\mu _c}''\varPsi _{e,c}'{{\hat{\mathfrak {q}}}}) \end{bmatrix},\nonumber \\ \end{aligned}$$

the bilinear operator is given as

2.8 $$\begin{aligned} B(\zeta ,\eta )=\frac{1}{2}\begin{bmatrix} a(\psi _{c,\mu _c}'')\left[ K^{-1}(\zeta _1\eta _1-\varPi ^{(2)}\zeta _1\eta _1+\zeta _1\eta _2)+\zeta _2K^{-1}\eta _1\right] \\ \frac{1}{l_c}(\psi _{c,\mu _c}'')(\zeta _2\eta _2+\varPi ^{(2)}\zeta _1\eta _1)\\ \frac{1}{4B^2l_c}(\mathcal {S}_{\mu _c}'')\zeta _3\eta _3 \end{bmatrix}, \end{aligned}$$

where $\zeta ,\eta \in U:={\mathcal {H}}\times {\mathbb {R}}\times {\mathbb {R}}$ and are written as $\zeta =[\zeta _1,\zeta _2,\zeta _3]$ and $\eta =[\eta _1,\eta _2,\eta _3]$. The trilinear operator is given as

2.9 $$\begin{aligned} {{\textbf {F}}}(v,v,v)=\frac{1}{6}\begin{bmatrix} a(\psi _c''')[K^{-1}(v_1^3-\varPi ^{(3)}v_3)+3K^{-1}(v_1^2v_2-\varPi ^{(2)}v_1^2v_2+v_1v_2^2)]\\ \frac{1}{l_c}(\psi _c''')(v_2^3+\varPi ^{(3)}v_1^3+3\varPi ^{(2)}v_1^2v_2)\\ \frac{1}{4B^2l_c}(\mathcal {S}_{\mu _c}''')v_3^3 \end{bmatrix},\nonumber \\ \end{aligned}$$

where $$v:=[v_1,v_2,v_3]\in U:={\mathcal {H}}\times {\mathbb {R}}\times {\mathbb {R}}$$ and $$\varPhi _{e,c}':=\varPhi _e'(\mu _c)$$, $$\varPsi _{e,c}':=\varPsi _e'(\mu _c)$$; $$\psi _{c,\mu }':=\psi _c'(\varPhi _e(\mu ))=\frac{3\iota }{2\varTheta }\left[ 1-\left( \frac{\varPhi _e(\mu )}{\varTheta }-1\right) ^2\right] $$, $$\mathcal {S}_{\mu }'=-\frac{\mu }{2\sqrt{\varPsi _e(\mu )}}$$; $$\psi _{c,\mu }'':=\psi _c''(\varPhi _e(\mu ))=-\frac{3\iota }{\varTheta ^2}(\frac{\varPhi _e(\mu )}{\varTheta }-1)$$, $$\mathcal {S}_{\mu }''=\frac{\mu }{4\sqrt{\varPsi _e(\mu )}^3}$$; $$\psi _c''':=\psi _c'''(\varPhi _e(\mu ))=-\frac{3\iota }{\varTheta ^3}$$, $$\mathcal {S}_{\mu }'''=-\frac{3\mu }{8\sqrt{\varPsi _e(\mu )}^5}$$.

The spectrum of $L({{\hat{\mathfrak {q}}}})$ Xiao (2008) in the neighborhood of $\mu _c$ is $\sigma (L({{\hat{\mathfrak {q}}}}))=\{ \lambda _{\pm n}({{\hat{\mathfrak {q}}}}), \gamma _{\pm 1}({{\hat{\mathfrak {q}}}}) \}$ for $n\in {\mathbb {Z}}^+$, where

2.10 $$\begin{aligned} \lambda _{\pm n}({{\hat{\mathfrak {q}}}})=\frac{a|n|}{|n|+am}\left( (\psi _{c,\mu _c}'+\psi _{c,\mu _c}''\varPhi _{e,c}'{{\hat{\mathfrak {q}}}})-\frac{\nu n^2}{2a}\pm \frac{|n|}{2a}i\right) \end{aligned}$$

for $n\in {\mathbb {Z}}^+$ and the corresponding eigenvectors are $\zeta _{\pm n}$ $=[e^{\pm in\theta },0,0]^\text {T}$;

$$\begin{aligned} \gamma _{\pm 1}({{\hat{\mathfrak {q}}}})=\frac{\chi ({{\hat{\mathfrak {q}}}})-\varXi ({{\hat{\mathfrak {q}}}})}{2}\pm i\frac{\sqrt{\frac{1}{B^2}-(\psi _{c,\mu _c}'-\frac{\mathcal {S}_{\mu _c}'}{4B^2})^2 }}{2l_c} \end{aligned}$$

where $\chi ({{\hat{\mathfrak {q}}}})= \frac{1}{l_c}(\psi _{c,\mu _c}'+\psi _{c,\mu _c}''\varPhi _{e,c}'{{\hat{\mathfrak {q}}}})$ and $\varXi ({{\hat{\mathfrak {q}}}})=-\frac{1}{4B^2l_c}(\mathcal {S}_{\mu _c}'+\mathcal {S}_{\mu _c}''\varPsi _{e,c}'{{\hat{\mathfrak {q}}}})$; the eigenvector corresponding to $\gamma _{\pm 1}({\hat{\mathfrak {q}}})$ is given by $\zeta _{\gamma _j}=\left[ 0,1,\zeta _{\psi _j}\right] ^\text {T}$ for $j\in \{\pm 1\}$, where $\zeta _{\psi _j}=\frac{l_c(\chi +\varXi )}{2}-ij\frac{\sqrt{\frac{1}{B^2}-(\psi _{c,\mu _c}'-\frac{\mathcal {S}_{\mu _c}'}{4B^2})^2 }}{2}.$ Based on (2.6), we can separate the slow and fast dynamics. For completeness, we state the other basic properties of the linear operator $L({\hat{\mathfrak {q}}})$ in Appendix A.

#### Remark 5

We also denote *B*(*v*, *v*) and ${{\textbf {F}}}(v,v,v)$ by *B*(*v*) and ${{\textbf {F}}}(v)$ for short.

### Projection and Simplifications

In this subsection, we provide the critical and stable dynamics for the stall case. A similar procedure can be used to study the surge as well as the stall-surge cases.

Let $\zeta :=\zeta _1$ and ${\overline{\zeta }}:=\zeta _{-1}$ (recall $\zeta _{\pm 1}$ in Sect. A-5) denote the critical eigenvectors. Then the corresponding adjoint eigenfunctions are $\zeta ^*:=[\frac{K^{-1}}{2\pi } e^{-i\theta },0,0]^T$ and ${\overline{\zeta }}^*:=[\frac{K^{-1}}{2\pi }e^{i\theta },0,0]^T$, and the corresponding eigenvalues are $\lambda _{\pm 1}({\hat{\mathfrak {q}}})$. Note that by the definition of inner product in Remark 3, we have $\langle \zeta ,\zeta ^* \rangle _U=1$, $\langle \zeta ,{\overline{\zeta }}^* \rangle _U =0$; $\langle {\overline{\zeta }},\zeta ^* \rangle _U =0$, $\langle {\overline{\zeta }},{\overline{\zeta }}^* \rangle _U =1$. The critical projection operator is explicitly defined by $P_c:=\langle \zeta ^*, \cdot \rangle _{U} \zeta +\langle {\overline{\zeta }}^*, \cdot \rangle _{U} {\overline{\zeta }}$, and the stable projection $P_s=I-P_c$. In particular, we use simple notations for the amplitudes of the critical projection, ${\hat{B}}:=\langle \zeta ^*, B\rangle _{U}$ as well as $\hat{{{\textbf {F}}}}:=\langle \zeta ^*, {{\textbf {F}}}\rangle _{U}$. We also denote $-L_{s}({\hat{\mathfrak {q}}}):=L({\hat{\mathfrak {q}}})$ when $L({\hat{\mathfrak {q}}})$ is restricted to $P_sU$, where the negative sign is to emphasize the sign of the stable eigenvalues.

We represent the solution $v\in U$ as $v=x+y$ for $x\in P_cU$ and $y\in P_sU$. By the above separation of spectrum, we obtain the critical and stable dynamics as:

2.11a $$\begin{aligned}&dz=\langle \zeta ^*, dx\rangle _U= \left[ \lambda _1({\hat{\mathfrak {q}}})z + \hat{B}(x+y,x+y) +\hat{{\textbf {F}}}(x+y,x+y,x+y)\right] dt; \end{aligned}$$

2.11b $$\begin{aligned}&dy=\left[ -L_s({\hat{\mathfrak {q}}})y+P_s B(x+y,x+y)+P_s {{\textbf {F}}}(x+y,x+y,x+y)\right] dt. \end{aligned}$$

where the amplitudes $z,{\overline{z}}\in {\mathbb {C}}$, $U_1^c\ni x=z\zeta +{\overline{z}}\overline{\zeta }$ and $P_sU_1 \ni y=v-x=[\sum _{n\in {\mathbb {Z}}{\setminus }\{0,\pm 1\}}g_ne^{in\theta },\varPhi _\delta ,\varPsi _\delta ]^{\text {T}}$. It is clear that $\text {Re}(\lambda _{\pm 1}({\hat{\mathfrak {q}}}))$ is linear in ${\hat{\mathfrak {q}}}$ (see the definition in (2.10)). Since *z* and $\overline{z}$ are conjugated conterparts, showing the dynamics of either one of them is sufficient to represent the critical dynamics.

Note that $P_c$ can be interpreted as a two-fold projection:

- projection from *U* onto ${\mathcal {H}}$;
- projection from ${\mathcal {H}}$ onto $U^{\text {stall}}_c$.

Furthermore,

1. $\hat{B}(x,x)=\langle \zeta ^*, B(x,x)\rangle _U=\langle \zeta ^*,B(z\zeta ,z\zeta )+2B(z\zeta ,{\overline{z}}{\overline{\zeta }})+B({\overline{z}}{\overline{\zeta }},{\overline{z}}{\overline{\zeta }})\rangle _U$, but we can justify that $\langle \zeta ^*,B(z\zeta ,z\zeta )\rangle _U=\langle \zeta ^*,B({\overline{z}}{\overline{\zeta }},{\overline{z}}{\overline{\zeta }})\rangle _U=\langle \zeta ^*,B(z\zeta ,{\overline{z}}{\overline{\zeta }})\rangle _U=0$
2. $$\hat{B}(y,y)=\langle \zeta ^*, B(y,y)\rangle _U=\frac{a(\psi _{c,\mu _c}'')}{1+am}\sum _{k\in \{-2,-3, \ldots \}}^{k+l=1}g_kg_l$$.
3. $$\hat{B}(x+y,x+y)=2\hat{B}(x,y)+\hat{B}(y,y)$$; $$P_cB(x+y,x+y)=2P_cB(x,y)+P_cB(y,y)$$.
4. $$P_sB(x+y,x+y)=B(x,x)+2P_sB(x,y)+P_sB(y,y)$$.

## Notations and Assumptions for Stochastic Moore-Greitzer Model

Based on (2.6), the main purpose of this paper is to investigate the dominating dynamics in the critical subspace of stall in the neighbourhood of $\mu _c$ and ${\hat{v}}=0$ with the presence of additive noise. In order to examine the behavior of the small solutions ${\hat{v}}(t):=\varepsilon ^{-1}v(\varepsilon ^{-2}t)$ of (2.6), we consider the following Cauchy problem

3.1 $$\begin{aligned} d{\hat{v}} =\varepsilon ^{-2}L({\hat{\mathfrak {q}}}){\hat{v}}dt+\varepsilon ^{-1}B({\hat{v}},{\hat{v}})dt+\text {F}({\hat{v}},{\hat{v}},{\hat{v}})dt+\varepsilon ^{-1} d{\hat{W}}_{t},\;\;\;{\hat{v}}(0)=\varepsilon ^{-1}{\hat{v}}_0,\nonumber \\ \end{aligned}$$

where ${\hat{W}}_t:=\varepsilon W_{\varepsilon ^{-2}t}$ and the semigroup^1^ associated to (3.1) is $\hat{S}(t)=e^{\varepsilon ^{-2}L({\hat{\mathfrak {q}}})t}$. In order to define the space-time model of $W_{t}$ and the solutions to SPDEs, it is necessary to set up spaces and assumptions such that the problem is well defined.

Note that based on the abstract form (3.1), $L({\hat{\mathfrak {q}}}):=A+Df_{u_e}({\hat{\mathfrak {q}}})$ keeps all the properties as introduced in Sect. A.

The solution space for the deterministic case can be found in Sect. A, where ${\mathcal {D}}(L({\hat{\mathfrak {q}}}))$ coincided with $H^2_{0}\times {\mathbb {R}}\times {\mathbb {R}}$. Now we define the fractional spaces w.r.t. ${\mathcal {D}}(L({\hat{\mathfrak {q}}}))$ and $H^2_{0}$ (Def. 6) for the stochastic settings in order to have a more flexible scale of regularity.

### Definition 6

(Fractional Power Space) For $\alpha \in {\mathbb {R}}$, given the analytic semigroup $\hat{S}(t)$ generated by $\varepsilon ^{-2}L({\hat{\mathfrak {q}}})$, define the interpolation fractional power (Hilbert) space Pazy (2012) $U_{\alpha }:={\mathcal {D}}(L^{\alpha }({\hat{\mathfrak {q}}}))$ endowed with inner product $\langle u,v\rangle _{\alpha }= \langle L^{\alpha }u,L^{\alpha }v\rangle _{U}$ and corresponding induced norm $\Vert \cdot \Vert _{\alpha }:=\Vert L^{\alpha }\cdot \Vert$. Similarly, as short-hand notation we define $L|_{{\mathcal {H}}}^\alpha :=(L|_{{\mathcal {H}}})^\alpha$ and ${\mathcal {H}}_{\alpha }:={\mathcal {D}} (L|_{{\mathcal {H}}}^{\alpha })$. Furthermore, the spaces $U_{\alpha }$ (resp. ${\mathcal {H}}_{\alpha }$) and $U_{-\alpha }$ (resp. ${\mathcal {H}}_{-\alpha }$) are dual to each other under the duality pairing w.r.t. $\langle \cdot ,\cdot \rangle _U$ (resp. $\langle \cdot ,\cdot \rangle _\mathcal {H}$).

### Remark 7

For the Moore-Greitzer model, due to (A.1), ${\mathcal {D}} (L|_{{\mathbb {R}}^2}^{\alpha }({\hat{\mathfrak {q}}}))$ is isomorphic to ${\mathbb {R}}^2$ (since there is no spatial dependence in this subspace), and therefore $U_{\alpha }$ is isomorphic to ${\mathcal {H}}_{\alpha }\times {\mathbb {R}}\times {\mathbb {R}}$.

We list other properties Hairer (2009) of the fractional power and $e^{Lt}$:

1. ${\mathcal {H}}_{\alpha }\subset {\mathcal {H}}_{\beta }$ for $\alpha \ge \beta$. Furthermore, for $\gamma >0$, ${\mathcal {H}}_{\gamma }\subset {\mathcal {H}}\subset {\mathcal {H}}_{-\gamma }$;
2. The quantity $e^{Lt}$ commutes with any power of its generator;
3. $\Vert P_sL^{\alpha }e^{Lt}\Vert \le \frac{C_{\alpha }}{t^{\alpha }}e^{-\omega t}$ for all $t>0$. In particular, $\Vert L^{\alpha }e^{Lt}\Vert \le \frac{C_{\alpha }}{t^{\alpha }}$ when $t\in (0,1]$.

### Proposition 8

For $\alpha >\beta \in {\mathbb {R}}$, $e^{Lt}$ maps ${\mathcal {H}}_{\beta }$ into ${\mathcal {H}}_{\alpha }$, there exists a constant $C_{\alpha ,\beta }$ such that for $t\in (0,1]$, $\Vert e^{Lt}x\Vert _{\alpha }\le C_{\alpha ,\beta }\Vert x\Vert _{\beta }t^{\beta -\alpha }$. Moreover, for all $t>0$ and $x\in P_sU$, there exists a constant $C_{\alpha ,\beta }'$ such that $\Vert e^{-L_st}x\Vert _{\alpha }\le C_{\alpha ,\beta }'\Vert x\Vert _{\beta }t^{\beta -\alpha }e^{-\omega t}$.

### Proof

For $t\in (0,1]$, we have

$$\begin{aligned} \Vert e^{Lt}x\Vert _{\alpha }=\Vert L^{\alpha }e^{Lt}x\Vert =\Vert L^{\alpha -\beta }e^{Lt}(L^{\beta })x\Vert \le \Vert L^{\alpha -\beta }e^{Lt}\Vert \Vert x\Vert _{\beta }, \end{aligned}$$

and by Remark 7,

$$\begin{aligned} \Vert L^{\alpha -\beta }e^{Lt}\Vert \le C t^{-\alpha +\beta }. \end{aligned}$$

we obtain the relation for $t\in (0,1]$. For general $t>0$, consider the stable projection, the part $e^{-\omega t}$ is inherited from the property of $e^{-L_st}$ (see in Sect. A-2). $\square$

### Definition 9

(Fractional Sobolev Space) We work with standard $L^2$-Sobolev spaces: Let $L^2 = L^2 ([0, 2 \pi ])$ be the space of square-integrable functions on $[0, 2 \pi ]$. Any $f \in L^2$ has a Fourier expansion $f = \sum _{k \in {\mathbb {Z}}} e^{i k \cdot } {\mathfrak {f}}_k$ with $\sum _k | {\mathfrak {f}}_k |^2 = 2 \pi \Vert f \Vert _{L^2}^2 < \infty$. We define

$$\begin{aligned} H^r :=\left\{ f = \sum _{k \in {\mathbb {Z}}} e^{i k \cdot } {\mathfrak {f}}_k: \Vert f \Vert _{H^r}^2 :=\sum _{k \in {\mathbb {Z}}} (1 + | k |^2)^r | {\mathfrak {f}}_k |^2 < \infty \right\} , \end{aligned}$$

where for $r \ge 0$ the series converges in $L^2$, and for $r < 0$ it is a formal Fourier series which converges as a distribution acting on $C^{\infty } ({\mathbb {R}}/ (2 \pi {\mathbb {Z}}))$, the space of infinitely smooth $2 \pi$-periodic functions. The spaces $H^r$ and $H^{- r}$ are dual to each other under the duality pairing $_{H^{- r}} \langle f, h \rangle _{H^r} = \frac{1}{2 \pi } \sum _{k \in {\mathbb {Z}}} {\mathfrak {f}}_k \overline{{\mathfrak {h}}_k}.$ We mostly work on the subspace

$$\begin{aligned} H^r_0 :=\{ f \in H^r: {\mathfrak {f}}_0 = 0 \}. \end{aligned}$$

It can be seen from the special case when $r=1$, ${\mathcal {D}}(L|_{{\mathcal {H}}}^{r})=H_{0}^{2r}$. In the following lemma, we show that such relation holds for any $r\in {\mathbb {N}}$. For completeness, we provide the proof of Lemma 10 in Appendix B. The results can be further extended when $H_{0}^{2r}$ is defined for negative and non-integer *r*.

### Lemma 10

On the spatial domain $D=[0,2\pi ]$, the Sobolev norm $\Vert \cdot \Vert _{H^r}$ is equivalent as the fractional power norm $\Vert \cdot \Vert _{r/2}$ for $r\in {\mathbb {N}}$.

*H*-valued Wiener processes are essential to the study of SPDEs, where *H* is referred as a general class of separable Hilbert spaces with complete orthonormal systems $\{e_k\}$. However, in practice (see Def. 11 and 13), it is convenient to find a proper space where the covariance operator *Q* is of trace class (trace of *Q* is finite), such that the noise can be constructed through a series expansion.

### Definition 11

(*Q*-Wiener Processes) Given a probability space $(\varOmega , {\mathscr {F}},{\mathbb {P}})$, let *H* be a separable Hilbert space with complete orthonormal systems $\{e_k\}$, let *Q* be a trace class nonnegative operator on *H*. An *H*-valued stochastic process $\{W_t\}_{t\ge 0}$ (also written as *W*) is called a Q-Wiener process if

(i) *W* has continuous trajectories ${\mathbb {P}}$-a.s. and $W_0=0$,
(ii) *W* has independent increments and the law satisfies
  $$\begin{aligned} {\mathscr {L}}(W_{t}-W_s)={\mathscr {N}}(0,(t-s)Q),\qquad t\ge s\ge 0, \end{aligned}$$

### Proposition 12

The covariance operator of an *H*-valued Q-Wiener process *W* can be expressed as $Q:=\sum q_k e_k\otimes e_k$, where $\{q_k\}$ is the point spectrum of *Q*.

### Definition 13

(Generalized *Q*-Wiener Processes) Let *H* be the same space as in Def. 11, let *W* be a Wiener process with covariance operator *Q*. Let $H_1$ be a Hilbert space such that $Q^{1/2}H$ is embedded into $H_1$ with a Hilbert-Schmidt embedding and *Q* is a trace class operator when extended to $H_1$. Then *W* is an $H_1$-valued *Q*-Wiener process, we also call *W* a generalized *Q*-Wiener process based on *H*. In particular, when $Q=I$, *W* is an $H_1$-valued cylindrical Wiener process (or a generalized cylindrical Wiener process based on *H*).

The viscous Moore-Greitzer equation is based on the Navier-Stokes equation and a non-rigorous stochastic homogenization theory of fluids Hou (2002). Even though it is not clearly understood how the noise can be introduced into the periodic turbulent flow $g\in {\mathcal {H}}$, we construct ${\mathcal {H}}_{\alpha }$-valued Q-Wiener processes as explained in Def. 11 and 13 using expansion for the specific model of the engine disturbances. Such a model naturally captures the average phenomena and satisfies the prior belief of the space-time disturbances.

### Definition 14

(Model of disturbances) For the Moore-Greitzer model, we restrict attention to ${\mathcal {H}}$ and construct,

3.2 $$\begin{aligned} W|_{{\mathcal {H}}}(t)\!=\!\sum \limits _{k\in {\mathbb {Z}}^+\setminus \{1\}}\sqrt{q_k}(\beta _k(t)+i\beta _{-k}(t))e^{ik\theta }\!+\!\sum \limits _{k\in {\mathbb {Z}}^-\setminus \{-1\}}\sqrt{q_k}(\beta _{-k}(t)-i\beta _{k}(t))e^{ik\theta }\nonumber \\ \end{aligned}$$

where $q_k=|k|^{-(4\alpha +1)-\upsilon }$ for any fixed $\upsilon >0$, $\beta _k(t)$ are i.i.d. ${\mathscr {F}}_{t}$-Brownian motions. Then the process $W|_{{\mathcal {H}}}$ belongs to ${\mathcal {H}}_{\alpha }$ a.s..

The following examples are special cases of the engine disturbances:

(i) (White in time, colored in space) when $\alpha \ge 0$, $q_k$ decays as *k* increases, then *Q* is a trace class operator (i.e. ${\text {Tr}}(Q)=\sum _{k\in {\mathbb {Z}}{\setminus }\{0,\pm 1\}} q_k<\infty$ Da Prato and Zabczyk (2014)) in ${\mathcal {H}}_{\alpha }\subset {\mathcal {H}}$, and $W|_{{\mathcal {H}}}$ is automatically an ${\mathcal {H}}$-valued *Q*-Wiener process;
(ii) (Space-time white noise) when $\alpha = -1/4-\upsilon /4$, $Q=I$, ${\text {Tr}}(Q)=\infty$ and (3.2) does not converge in ${\mathcal {H}}$. However, when ${\mathcal {H}}$ is extended to ${\mathcal {H}}_{\alpha }\supset {\mathcal {H}}$ by a Hilbert-Schmidt inclusion operator, the $W|_{{\mathcal {H}}}$ is well defined as an ${\mathcal {H}}_{\alpha }$-valued process.

The construction (3.2) implies $\langle Q\zeta _k,\zeta _k^*\rangle =q_k=0$ for $k\in \{1,-1\}$, which means that the additive noise does not act on $P_c{\mathcal {H}}$. This is, in that, the additive stochastic components in the stable, heavily damped modes also contribute to the critical modes. These contributions enter the critical modes as multiplicative noise. If additional additive noise is acting directly on the critical modes, it will be of higher order than the multiplicative effects generated by the interaction between critical and stable modes. However, the stochastic stability of the fixed point is only affected by the presence of multiplicative noise in the critical modes. The proposed model of disturbances eliminates this strong additive effect to better understand and quantify the bifurcation behavior.

### Assumption 15

Given the probability measure space $(\varOmega , {\mathscr {F}},{\mathbb {P}})$, for $\alpha \in (\frac{1}{12},1]$, let $W_{t}=[W|_{{\mathcal {H}}}(t),\beta _{\varPhi }(t),\beta _{\varPsi }(t)]$ where $W|_{{\mathcal {H}}}(t)$ is a generalized Q-Wiener process constructed by (3.2), $\beta _{\varPhi }(t)$ and $\beta _{\varPsi }(t)$ are i.i.d. ${\mathscr {F}}_t$-Brownian motions in ${\mathbb {R}}$. We assume that there exists some (small) $\upsilon >0$ such that

3.3 $$\begin{aligned} {\Vert Q^{1/2}L|_{{\mathcal {H}}}^{\alpha -\frac{1}{2}+\upsilon }u\Vert <\infty ,\;\;u\in {\mathcal {H}}.} \end{aligned}$$

### Lemma 16

For any $\alpha >\frac{1}{12}$ there exists $\beta \in (\alpha -1,\alpha ]$ such that $B: U_{\alpha }\otimes U_{\alpha }\rightarrow U_{\beta }$ and ${{\textbf {F}}}:U_{\alpha }\otimes U_{\alpha }\otimes U_{\alpha }\rightarrow U_{\beta }$ are bounded multilinear operators.

### Proof

In this proof only we make use of more general Besov spaces than $H^r$, see Bahouri et al. (2011) for details. As $\mathcal {H}_\alpha \cong H_0^{2\alpha }$ is continuously embedded in $B^{2\alpha -\frac{1}{2}}_{\infty ,2}$ and $2\alpha +2\alpha >0$, standard multiplication results in Besov spaces (Bahouri et al. 2011, Theorem 2.82, Theorem 2.85) show that $\mathcal {H}_\alpha \times \mathcal {H}_\alpha \ni (u,v)\mapsto uv \in H^{(4\alpha - \frac{1}{2})\wedge 2\alpha }$ is a bounded bilinear operator. Moreover, in *B* also the 0 Fourier mode is removed and therefore we can take $\beta =(2\alpha - \frac{1}{4})\wedge \alpha \ge \alpha - \frac{1}{4}$ for this term. Repeating the argument and using that $4\alpha - \frac{1}{2} + 2\alpha > 0$, we get that $\mathcal {H}_\alpha ^3 \ni (u,v,w)\mapsto uvw \in H^{(6\alpha - 1)\wedge 2\alpha }$ is a bounded trilinear operator, so after removing the zero Fourier mode we can take $\beta = (3\alpha - \frac{1}{2})\wedge \alpha \ge \alpha - \frac{1}{2}$ for the ${{\textbf {F}}}$ term.

### Proposition 17

Suppose that Assumption 15 holds, then for each ${\mathfrak {q}}\in {\mathbb {R}}$ and ${\hat{v}}(0)\in U_\alpha$, (3.1) has a unique local mild solution ${\hat{v}}\in C([0,\tau _\infty );U_\alpha )$ of the form

3.4 $$\begin{aligned} {\hat{v}}(t)= & {} \hat{S}(t){\hat{v}}_0+\int _0^{t}\hat{S}(t-s)[\varepsilon ^{-1}B+{{\textbf {F}}}]({\hat{v}})ds\nonumber \\{} & {} +\varepsilon ^{-1}\int _0^{t}\hat{S}(t-s)dW_s, \;t\in (0,\tau _\infty ), \mathbb {P}\text {-a.s.} \end{aligned}$$

The stopping time is such that $\tau _{\infty }>0$ a.s. and satisfies $\lim _{t\rightarrow \tau _{\infty (\omega )}}\Vert {\hat{v}}(t)\Vert _\alpha =\infty$ or $\tau _\infty (\omega )=\infty$.

### Proof

We show a sketch of the proof based on the standard procedure. More examples can be found in Mohammed et al. (2014), Blömker and Romito (2015), Ball (1982). For the Moore-Greitzer model, by Lemma 16 and Proposition 8, it can be easily shown that $\Vert \hat{S}(t-s)B({\hat{v}})\Vert _\alpha$ and $\Vert \hat{S}(t-s){\textbf{F}}({\hat{v}})\Vert _\alpha$ exist. On the other hand, denoting the stochastic convolution term as $W_{\hat{S}}(t):=\int _0^t \hat{S}(t-s)dW_s$, by isometry and Assumption 15, we have

3.5 $$\begin{aligned} \begin{aligned} {\mathbb {E}}\left[ \left\| W_{\hat{S}(t)}\right\| _{\alpha }^2\right]&\le \int _0^t\Vert Q^{1/2}L|_{{\mathcal {H}}}^{\alpha }\hat{S}(t-s)\Vert _{{\mathcal {L}}_2(\mathcal {H},\mathcal {H})}^2ds+\int _0^t \Vert L|_{{\mathbb {R}}^2}^\alpha \hat{S}(t-s)\Vert ^2 ds\\&\le \Vert Q^{1/2}L|_{{\mathcal {H}}}^{\alpha -\frac{1}{2}+\upsilon }\Vert _{{\mathcal {L}}_2(\mathcal {H},\mathcal {H})}^2\int _0^t\Vert L|_{{\mathcal {H}}}^{1/2-\upsilon }\hat{S}(t-s)\Vert _{{\mathcal {L}}_2(\mathcal {H},\mathcal {H})}^2ds\\&\quad +\int _0^t \Vert L|_{{\mathbb {R}}^2}^\alpha \hat{S}(t-s)\Vert ^2 ds<\infty , \end{aligned} \end{aligned}$$

where $\Vert \cdot \Vert _{{\mathcal {L}}_2(\mathcal {H},\mathcal {H})}$ stands for the Hilbert-Schmidt norm. The stochastic convolution $W_{\hat{S}}$ is hence an Ornstein-Uhlenbeck process that takes values in $U_\alpha$ for all $t>0$. The local existence of the solution follows a standard procedure. One can investigate the quantity $h={\hat{v}}-\varepsilon ^{-1}W_{\hat{S}}$ pathwisely and treat $\varepsilon ^{-1}W_{\hat{S}}$ as a perturbation. The pathwise uniqueness up to some $\tau _\infty (\omega )$ is determined by the local Lipschitz continuity of *B* and ${\textbf{F}}$. In particular, the nonlinearities do not possess dissipativity, the pathwise global existence of the solution processes may not be guaranteed.

We also need to specify the stopping time, such that the approximation processes will stop before the solution ${\hat{v}}(t)$ blows up.

### Definition 18

(Stopping time) Given the terminal time *T* for (3.4) and a fixed $\kappa >0$, consider the stopping time

$$\begin{aligned} \tau ^*:= T\wedge \inf \{t>0: \;\Vert {\hat{v}}(t)\Vert _{\alpha }\ge \varepsilon ^{-\kappa }\}. \end{aligned}$$

### Definition 19

(Other notations) We introduce other notations for future references.

1. We specify the unfolding parameter to be $\hat{\mathfrak {q}}:=\varepsilon ^2\mathfrak {q}$ for some $\mathfrak {q}\in \mathbb {R}$.
2. For any state variable $\xi$, the quantity ${\hat{\xi }}(t)$ represents the value under scaling.
3. For the critical mode, let $\lambda _{\pm 1}({\mathfrak {q}})=\alpha _c(\mathfrak {q})\pm i\omega _c(\mathfrak {q}):={\mathfrak {q}}\alpha _1'(\mu _c)\pm i\varepsilon ^{-2} \omega _1(\varepsilon ^{2}{\mathfrak {q}})$ denote the eigenvalues^2^.
4. For the stable modes, $-L_{s}({\mathfrak {q}}):=L|_{P_sU}({\mathfrak {q}})$ (*L* restricted to $P_sU$). Without loss of generality, we let $L_s:=L_s(0)$, let $\lambda _k^s:=\alpha _k^s+i\omega _k^s$ be the eigenvalues of $-L_s$ for $k\in {\mathbb {Z}}{\setminus }\{0,\pm 1\}$, let the perturbation be $L_p({\mathfrak {q}}):=L_s({\mathfrak {q}})-L_s$. It is clear that $\lambda _k^s$’s are constants and $L_p({\mathfrak {q}})$ is linear in ${\mathfrak {q}}$.
5. We symbolically represent the inverse operator in ${\mathbb {R}}^2$ that defines $(\varPhi _\delta ,\varPsi _\delta )$ as
  3.6 $$\begin{aligned} -L_s|_{{\mathbb {R}}^2}^{-1}:=\begin{bmatrix} l_{11} &{} l_{12}\\ l_{21} &{} l_{22} \end{bmatrix}, \end{aligned}$$
  where $$l_{ij}\in {\mathbb {R}}$$ for $$i,j\in \{1,2\}$$.
6. Let
  $$\begin{aligned} -L_s^{\varepsilon }:=-L_s({\hat{\mathfrak {q}}})=-L_s-\varepsilon ^2L_p({\mathfrak {q}}). \end{aligned}$$
7. For $n\in {\mathbb {Z}}\setminus \{0,\pm 1\}$, ${\tilde{y}}=[\sum _n{\tilde{g}}_n e^{in\theta }, \;{\tilde{\varPhi }}_\delta , {\tilde{\varPsi }}_\delta ]^{\text {T}}$ denotes the solution to
  $$\begin{aligned} d{\tilde{y}}(t)=-\varepsilon ^{-2}L_s{\tilde{y}}dt+\varepsilon ^{-1}P_sdW_t,\;\;\;{\tilde{y}}(0)=\hat{y}(0), \end{aligned}$$
  and $y^*=\left[ \sum _n g^*_n e^{in\theta }, \;\varPhi ^*_\delta , \varPsi ^*_\delta \right] ^T$ denotes the associated stationary solution.
8. For convenience, we introduce
  3.7 $$\begin{aligned} {\mathcal {K}}_i=\frac{a\psi _{c,\mu _c}''|i|}{|i|+am}\;\;\text {for}\;\; i\in {\mathbb {Z}}\setminus \{0\} \end{aligned}$$
  and
  3.8 $$\begin{aligned} {\mathcal {G}}_i=\frac{a\psi _{c,\mu _c}''|i|}{2(|i|+am)}\left( 2({\hat{\varPhi }}_\delta )g_i+\sum ^{j=i-h}_{h\in {\mathbb {Z}}\setminus \{\pm 1\}}{\hat{g}}_{h}{\hat{g}}_{j}\right) \;\;\text {for}\;\;i\in {\mathbb {Z}}\setminus \{0,\pm 1\} \end{aligned}$$

## Dimension Reduction of Stochastic Moore-Greitzer Model

As introduced in Def. 14 and Assumption 15, the Q-Wiener process $W\in U_{\alpha }$ can be represented as

4.1 $$\begin{aligned} W=[W|_{{\mathcal {H}}}, \beta _{\varPhi },\beta _{\varPsi }]^{\text {T}}. \end{aligned}$$

### Finite-Dimensional Reduction of Dynamics for ${\hat{v}}$

We proceed as in Sect. 2.2 for (3.1) with the scaling ${\hat{\mathfrak {q}}}:=\varepsilon ^{2} {\mathfrak {q}}$ to obtain $\hat{x}=\hat{z}\zeta + {\overline{\hat{z}}}\overline{\zeta }$ and ${\hat{v}}(t)= \hat{z}(t)\zeta +{\overline{\hat{z}}}(t)\overline{\zeta }+ \hat{y}(t)$. When the system is close to the critical point, the local critical and fast-varying stable dynamics are as follows:

4.2a $$\begin{aligned}&d\hat{z}= \left[ \lambda _1({\mathfrak {q}})\hat{z}+2\varepsilon ^{-1}\hat{B}(\hat{x},\hat{y})+\varepsilon ^{-1}\hat{B}(\hat{y},\hat{y})+ \hat{{\textbf {F}}}(\hat{x}+\hat{y})\right] dt,\;\;\hat{z}(0)=\langle \nu ^*,{\hat{v}}(0)\rangle _U \end{aligned}$$

4.2b $$\begin{aligned}&d\hat{y}=\left[ -\varepsilon ^{-2}L_{s}^{\varepsilon }\hat{y}+\varepsilon ^{-1}P_s B(\hat{x}+\hat{y})+P_s {{\textbf {F}}}(\hat{x}+\hat{y})\right] dt+\varepsilon ^{-1} P_sdW_t,\;\hat{y}(0)=P_s{\hat{v}}(0) \end{aligned}$$

where $-L_s^{\varepsilon }:=-L_s(\varepsilon ^2{\mathfrak {q}})=-L_s-\varepsilon ^2L_p({\mathfrak {q}})$ as introduced in Def. 19.

Note that (4.2a), which provides dominant dynamics, has no explicit dependence on the stochastic perturbations. To obtain a finite-dimensional approximation for ${\hat{v}}$ based on (4.2a), we first investigate how the fast-varying $\hat{y}$, which contains the stochastic terms, enters the terms of intermediate order ${\mathcal {O}}(\varepsilon ^{-1})$. The approach follows the idea provided in (Blömker et al. (2007), Proposition 3.9). We provide the proof explicitly considering the complexity of the product state space *U*.

#### Lemma 20

For every stopping time $\sigma \le \tau ^*$, we have

4.3 $$\begin{aligned} \begin{aligned} \int _0^{\sigma }B(\hat{x},\hat{y})dt&=\varepsilon \int _0^{\sigma } B(\hat{x},L_{s}^{-1}P_sB(\hat{x}+\hat{y}))dt+2\varepsilon \int _0^{\sigma } B(P_cB(\hat{x},\hat{y}),\;L_{s}^{-1}\hat{y})dt\\&\quad +\varepsilon \int _0^{\sigma }B(P_cB(\hat{y},\hat{y}),\;L_{s}^{-1}\hat{y})dt+\varepsilon \int _0^{\sigma }B(\hat{x},L_{s}^{-1}P_sdW_t)+{\mathcal {O}}(\varepsilon ^2) \end{aligned} \end{aligned}$$

#### Proof

Expand the Q-Wiener process as (4.1), then

$$\begin{aligned} P_sW_t=\left[ 2\sum \limits _{k\in {\mathbb {Z}}^+\setminus \{1\}}\sqrt{ q_k}(\beta _k(t)cos(k\theta )-\beta _{-k}(t)sin(k\theta )),\beta _{\varPhi }(t),\beta _{\varPsi }(t)\right] ^T \end{aligned}$$

Now apply the infinite-dimensional Itô’s formula,

$$\begin{aligned} dB(\hat{x},L_{s}^{-1}\hat{y})=B(d\hat{x},L_{s}^{-1}\hat{y})+B(\hat{x},L_{s}^{-1}d\hat{y})+\frac{1}{2}\sum _{i,j}\frac{\partial ^2 B(\hat{x},L_{s}^{-1}\hat{y})}{\partial u_i\partial u_j}d\langle U_i,U_j\rangle \end{aligned}$$

where $i,j\in \{1,2\}$, $U_1=\hat{x}$, $U_2=\hat{y}$, and $d\langle \beta _k,\beta _l\rangle =\delta _{kl}dt$, $d\langle \beta _k,t\rangle =d\langle t,\beta _k\rangle =0$ for all *k*, *l* in the index set ${\mathbb {Z}}^+\setminus \{1\}\cup \{\varPhi ,\varPsi \}$. However, $\frac{\partial ^2 B(\hat{x},L_{s}^{-1}\hat{y})}{\partial \hat{x}^2}=\frac{\partial ^2 B(\hat{x},L_{s}^{-1}\hat{y})}{\partial \hat{y}^2}=0$, and therefore

$$\begin{aligned} \frac{1}{2}\sum _{i,j}\frac{\partial ^2 B}{\partial u_i\partial u_j}d\langle U_i,U_j\rangle =\frac{1}{2}\left( B(d\hat{x},dL_{s}^{-1}\hat{y})+B(d\hat{x},dL_{s}^{-1}\hat{y}) \right) =B(d\hat{x},dL_{s}^{-1}\hat{y}) \end{aligned}$$

By plugging in $d\hat{x}$, $dL_{s}^{-1}\hat{y}$ and eliminating all the $d\beta _idt$, $dtd\beta _i$, *dtdt* terms,

$$\begin{aligned} \frac{1}{2}\sum _{i,j}\frac{\partial ^2 B(\hat{x},L_{s}^{-1}\hat{y})}{\partial u_i\partial u_j}d\langle U_i,U_j\rangle =B(dP_cdW_t,dP_sdW_t)=0 \end{aligned}$$

Hence,

$$\begin{aligned} \begin{aligned} \varepsilon ^2dB(\hat{x},L_{s}^{-1}\hat{y})&=\varepsilon ^2B(d\hat{x},L_{s}^{-1}\hat{y})+\varepsilon ^2B(\hat{x},L_{s}^{-1}d\hat{y})\\&=\varepsilon ^{2}B(\lambda _1\hat{z}\nu +\lambda _{-1}{\overline{\hat{z}}}\overline{\zeta },\;L_{s}^{-1}\hat{y})dt+2\varepsilon B(P_cB(\hat{x},\hat{y}),\;L_{s}^{-1}\hat{y})dt\\&\quad +\varepsilon B(P_cB(\hat{y},\hat{y}),\;L_{s}^{-1}\hat{y})dt+\varepsilon ^{2}B(P_c{{\textbf {F}}},\;L_{s}^{-1}\hat{y}))dt \\&\quad -B(\hat{x},\hat{y})dt+\varepsilon B(\hat{x},L_{s}^{-1}P_sB_1)dt+\varepsilon ^{2}B(\hat{x},P_sL_{s}^{-1}{{\textbf {F}}})dt \\&\quad -\varepsilon ^2B(\hat{x},L_{s}^{-1}L_{p}\hat{y})dt+\varepsilon B(\hat{x},L_{s}^{-1}P_sdW_t)+{\mathcal {O}}(\varepsilon ^3) \end{aligned} \end{aligned}$$

The result follows straightforwardly after this. In addition, the above terms contain the operation of the form $U_\alpha \otimes U_\beta$, where $\alpha ,\beta$ are as given in Lemma 16. By a similar technique, one can show that $U_\alpha \otimes U_\beta \rightarrow U_\gamma$ for some $\gamma \in (\alpha -1,\alpha ]$, and hence $\Vert S(t-s)u\Vert _\alpha <\infty$ for any $u\in U_\gamma$. $\square$

#### Lemma 21

For every stopping time $\sigma \le \tau ^*$, we have

4.4 $$\begin{aligned} \begin{aligned}&\int _0^{\sigma }\varepsilon ^{-1}\hat{B}(\hat{y},\hat{y})dt \\&\quad =-\int _0^{\sigma }{\mathcal {K}}_1\sum _{k\in \{-2,-3 \ldots \}}^{k+l=1}\left( \frac{ \hat{z}({\mathcal {K}}_k{\hat{g}}_k{\hat{g}}_{-k}+{\mathcal {K}}_l{\hat{g}}_l{\hat{g}}_{-l})}{\lambda _k^s+\lambda _l^s} dt+\frac{ {\hat{g}}_k{\mathcal {G}}_l+{\hat{g}}_l{\mathcal {G}}_k}{\lambda _k^s+\lambda _l^s}dt\right) \\&\quad =-\int _0^{\sigma }\frac{{\mathcal {K}}_{1}{\mathcal {K}}_{2}g_3\overline{\hat{z}}^2}{2(\lambda _{-2}^s+\lambda _3^s)} dt-\int _0^{\sigma }{\mathcal {K}}_1\sum _{k\in \{-2,-3 \ldots \}}^{k+l=1}\frac{ \overline{\hat{z}}({\mathcal {K}}_{l+1}{\hat{g}}_k{\hat{g}}_{l+1}+\int _0^{\sigma }{\mathcal {K}}_{k+1}{\hat{g}}_l{\hat{g}}_{k+1})}{\lambda _k^s+\lambda _l^s}dt\\&\qquad -\int _0^{\sigma }{\mathcal {K}}_1\sum _{k\in \{-2,-3 \ldots \}}^{k+l=1}\frac{{\hat{g}}_k\sqrt{q_l} (d\beta _{l}(t)+id\beta _{-l}(t))+{\hat{g}}_l\sqrt{q_k} (d\beta _{-k}(t)-id\beta _{k}(t))}{\lambda _k^s+\lambda _l^s}\\&\qquad +{\mathcal {O}}(\varepsilon ), \end{aligned} \end{aligned}$$

where ${\mathcal {K}}_i$,${\mathcal {G}}_i$ and $\lambda _i^s$ are defined in Def. 19.

#### Remark 22

The idea is the same as Lemma 20. However, for completeness, we provide the detailed proof in Appendix C.

Now by applying Lemma 20 and 21 to (4.2), we have the projected equations

4.5a $$\begin{aligned} d\hat{z}&= \left[ \lambda _1(\mathfrak {q})\hat{z}+ \hat{{\textbf {F}}}(\hat{x}+\hat{y})\right] dt+2\hat{B}(\hat{x},L_{s}^{-1}P_sB(\hat{x}+\hat{y}))dt \nonumber \\&\quad +4 \hat{B}(P_cB(\hat{x},\hat{y}),\;L_{s}^{-1}\hat{y})dt+2 \hat{B}(P_cB(\hat{y},\hat{y}),\;L_{s}^{-1}\hat{y})dt\nonumber \\&\quad + 2\hat{B}(\hat{x},L_{s}^{-1}P_sdW_t) +\varepsilon ^{-1}\hat{B}(\hat{y},\hat{y})dt+{\mathcal {O}}(\varepsilon )\end{aligned}$$

4.5b $$\begin{aligned} d\hat{y}&=\left( -\varepsilon ^{-2}L_s^{\varepsilon }\hat{y}+\varepsilon ^{-1}P_s B(\hat{x}+\hat{y})+P_s{{\textbf {F}}}(\hat{x}+\hat{y})\right) dt+\varepsilon ^{-1}P_sdW_t \end{aligned}$$

Note that in (4.5a), the term $\varepsilon ^{-1}{\hat{B}}(\hat{y},\hat{y})$ defined by (4.4) is indeed of order ${\mathcal {O}}(1)$. Hence, the amplitude equation (4.5a) is scaled such that the nonlinearities and the linear term are of the same order, which makes the analysis more amenable.

#### Remark 23

In the case of a surge bifurcation, we would have $\hat{B}(\hat{x},\hat{x})\ne 0$ with the same rescaling scheme. Since there is no contribution of homogenization from the stable modes, this term would dominate the rescaled critical mode with strength $\varepsilon ^{-1}$. Hence, to yield a similar form as (4.5), we should rescale the variables differently. One possibility would be to set ${\hat{z}}(t):=\varepsilon ^{-2}z(\varepsilon ^{-2}t)$ and ${\hat{y}}(t):=\varepsilon ^{-2}v(\varepsilon ^{-2}t)$. As for the stall-surge case, multiple rescaling schemes are needed to capture the bifurcation of ${\hat{g}}$ and $({\hat{\varPhi }}_\delta ,{\hat{\varPsi }}_\delta )$.

To keep this paper succinct, we only demonstrate the methodology via the stochastic analysis for the stall instability. The cases for surge and stall-surge can be treated using similar methods.

### Approximation of the Stable Modes

The purpose of this subsection is to find an approximation of the stable dynamics.

#### Lemma 24

Let ${\tilde{y}}(t)$ solve the Ornstein-Uhlenbeck equation

4.6 $$\begin{aligned} d{\tilde{y}}(t)=-\varepsilon ^{-2}{L_s}{\tilde{y}}dt+\varepsilon ^{-1}P_sdW_t,\;\;\;{\tilde{y}}(0)=\hat{y}(0), \end{aligned}$$

then ${\mathbb {E}}\sup _{0\le t\le \tau ^*}\Vert \hat{y}(t)-{\tilde{y}}(t)\Vert _{\alpha }={\mathcal {O}}(\varepsilon ^{1-2\kappa })$ for every $\kappa >0$ (see in Def. 18).

#### Proof

We spell out the proof for $\alpha >1/4$ (which implies that *B* and ${\textbf{F}}$ map from $U_\alpha$ to $U_\alpha$), and the proof for the rest of situation is similar. Let ${\hat{S}}_s(t):=e^{-\varepsilon ^{-2}{L_s} t}$. Note that $-{L_s}$ provides a stable spectrum, by Proposition 8, there exist $C>0$ and $\omega >0$ such that,

$$\begin{aligned} \Vert e^{-L_st}x\Vert _{\alpha }\le C\Vert x\Vert _{\alpha }e^{-\omega t}, \end{aligned}$$

hence,

$$\begin{aligned} \Vert {\hat{S}}_s(t)x\Vert _{\alpha }\le C\Vert x\Vert _{\alpha }e^{-\varepsilon ^{-2}\omega t}. \end{aligned}$$

Since

$$\begin{aligned} \hat{y}(t)-{\tilde{y}}(t)= & {} {\int _0^t{\hat{S}}_s(t-\sigma )L_p(\mathfrak {q})\hat{y}(\sigma )}d\sigma +\int _0^{t}{\hat{S}}_s(t-\sigma )[\varepsilon ^{-1}P_sB\\{} & {} \quad +P_s{{\textbf {F}}} ](\hat{x}(\sigma )+\hat{y}(\sigma ))d\sigma , \end{aligned}$$

together with the boundedness property of ${\hat{S}}_s$, for each $p>0$, we have

$$\begin{aligned} \begin{aligned}&{\mathbb {E}}\sup _{0\le t\le \tau ^*}\int _0^t\left[ \Vert {\hat{S}}_s(t-\sigma )\varepsilon ^{-1}P_sB(\hat{x}(\sigma )+\hat{y}(\sigma ))\Vert _\alpha d\sigma \right] ^p\\&\quad \le C \varepsilon ^{2p(\alpha -\beta )}{\mathbb {E}}\sup _{0\le t\le \tau ^*}\int _0^t\left[ e^{-\varepsilon ^{-2}\omega (t-\sigma )}(t-\sigma )^{\beta -\alpha }\Vert \varepsilon ^{-1}P_sB(\hat{x}(\sigma )+\hat{y}(\sigma ))\Vert _\beta d\sigma \right] ^p\\&\quad \le C\varepsilon ^{2p(\alpha -\beta )-p}{\mathbb {E}}\sup _{0\le t\le \tau ^*}\int _0^t\left[ e^{-\varepsilon ^{-2}\omega (t-\sigma )}(t-\sigma )^{\beta -\alpha }\Vert \hat{x}(\sigma )+\hat{y}(\sigma )\Vert _\alpha ^2 d\sigma \right] ^p\\&\quad \le C\varepsilon ^{p}{\mathbb {E}}\sup _{0\le t\le \tau ^*}\int _0^t\left[ e^{-\varepsilon ^{-2}\omega (t-\sigma )}(t-\sigma )^{\beta -\alpha }\Vert \hat{x}(\sigma )+\hat{y}(\sigma )\Vert _\alpha ^2 d\sigma \right] ^p\\&\quad \le C\varepsilon ^{p-2\kappa }\sup _{0\le t\le \tau ^*}\int _0^t\left[ e^{-\varepsilon ^{-2}\omega (t-\sigma )}(t-\sigma )^{\beta -\alpha } d\sigma \right] ^p={\mathcal {O}}(\varepsilon ^{1-2\kappa }). \end{aligned} \end{aligned}$$

The bounds for the other terms are obtained in a similar way. Combining the above, we have ${\mathbb {E}}\sup _{0\le t\le \tau ^*}\Vert \hat{y}(t)-{\tilde{y}}(t)\Vert _{\alpha }$ is of order ${\mathcal {O}}(\varepsilon ^{1-2\kappa })$.

#### Corollary 25

For all $t\in (0,\tau ^*]$, we have

$$\begin{aligned} \Vert B(\hat{y}(t),\hat{y}(t))-B({\tilde{y}}(t),{\tilde{y}}(t))\Vert _\alpha ={\mathcal {O}}(\varepsilon ^{1-2\kappa }) \end{aligned}$$

and

$$\begin{aligned} \Vert {\textbf{F}}(\hat{x}(t)+\hat{y}(t))-{\textbf{F}}(\hat{x}(t)+{\tilde{y}}(t))\Vert _\alpha ={\mathcal {O}}(\varepsilon ^{1-2\kappa }). \end{aligned}$$

#### Proof

$$\begin{aligned} \begin{aligned} \Vert B(\hat{y},\hat{y})-B({\tilde{y}},{\tilde{y}})\Vert _\alpha&= \Vert B(\hat{y},\hat{y})-B(\hat{y},{\tilde{y}})+B(\hat{y},{\tilde{y}})-B({\tilde{y}},{\tilde{y}})\Vert _\alpha \\&\le \Vert B(\hat{y},\hat{y}-{\tilde{y}})\Vert _\alpha +\Vert B(\hat{y}-{\tilde{y}},{\tilde{y}})\Vert _\alpha \end{aligned} \end{aligned}$$

By Lemma 24 and the boundedness of *B*, the result follows. The proof for ${\textbf{F}}$ is similar.

#### Remark 26

Due to the strong dissipativity of the semigroup generated by $-L_s$, it can be easily verified that the quantity ${\mathbb {E}}\sup _{0\le t<\tau ^*}\left\| \int _0^t\varepsilon ^{-2tL_s}dW_\sigma d\sigma \right\| _\alpha ^p$ is bounded, which implies that ${\mathbb {E}}\sup _{0\le t<\tau ^*}\Vert \tilde{y}(t)\Vert _\alpha ^p$ (resp. ${\mathbb {E}}\sup _{0\le t<\tau ^*}\Vert \hat{y}(t)\Vert _\alpha ^p$) is bounded by $C_p\varepsilon ^p$ for each $p>0$ and some $C_p$. The smallness of the stable mode as well as its approximation do not contribute much to the state explosion.

#### Corollary 27

By replacing $\hat{y}$ with ${\tilde{y}}$ in (4.5a), we have

4.7a $$\begin{aligned} d\hat{z}&= \left[ \lambda _1(\mathfrak {q})\hat{z}+ \hat{{\textbf {F}}}(\hat{x}+\tilde{y})\right] dt+2\hat{B}(\hat{x},L_{s}^{-1}P_sB(\hat{x}+\tilde{y}))dt \nonumber \\&\quad +4 \hat{B}(P_cB(\hat{x},\tilde{y}),\;L_{s}^{-1}\tilde{y})dt+2 \hat{B}(P_cB(\tilde{y},\tilde{y}),\;L_{s}^{-1}\tilde{y})dt\nonumber \\&\quad + 2\hat{B}(\hat{x},L_{s}^{-1}P_sdW_t) +\varepsilon ^{-1}\hat{B}(\tilde{y},\tilde{y})dt+{\mathcal {O}}(\varepsilon )\end{aligned}$$

4.7b $$\begin{aligned} d\tilde{y}&= -\varepsilon ^{-2}L_s\tilde{y}dt+\varepsilon ^{-1}P_sdW_t \end{aligned}$$

#### Proof

By iteratively using Corollary 25 on the nonlinearities, we see that the replacing error belongs to ${\mathcal {O}}(\varepsilon )$.

In order to study the long-term behavior of (4.5a), we would like to average out the fast modes $\hat{y}$ over an invariant measure by considering the stationary behavior of $y_s^*$ given by (4.5b). This is encapsulated in the homogenization procedure discussed in Sect. 5. However, based on Corollary 27, considering evaluating the solution ${\hat{z}}\in P_cU$ by the integral form, it will not cause any larger errors in the critical mode by using $y^*$ (that is, the stationary solution of (4.7b)) instead of ${\tilde{y}}$ on the R.H.S. of (4.7a).

## Approximation Equations

In this section, an explicit expression of $y^*$ is determined. Then by substituting $y^*$ into (4.7a), the dynamical behavior of the critical mode is studied.

### Calculation of $y^*$

Equation (4.6) can be decomposed into

5.1a $$\begin{aligned}&d{\tilde{g}}_k(t)=\varepsilon ^{-2}\lambda _k^{\varepsilon }{\tilde{g}}_kdt+\varepsilon ^{-1}\sqrt{q_k} (d\beta _k(t)+id\beta _{-k}(t)),\;\forall k\in \{2,3, \ldots \} \end{aligned}$$

5.1b $$\begin{aligned}&d{\tilde{g}}_k(t)=\varepsilon ^{-2}\lambda _k^{\varepsilon }{\tilde{g}}_kdt+\varepsilon ^{-1}\sqrt{q_k}(d\beta _{-k}(t)-id\beta _{k}(t)),\;\forall k\in \{-2,-3, \ldots \} \end{aligned}$$

5.1c $$\begin{aligned}&d\begin{bmatrix} {\tilde{\varPhi }}_\delta (t)\\ {\tilde{\varPsi }}_\delta (t) \end{bmatrix}=-\varepsilon ^{-2}L_s|_{{\mathbb {R}}^2}\begin{bmatrix} {\tilde{\varPhi }}_\delta (t)\\ {\tilde{\varPsi }}_\delta (t) \end{bmatrix}dt+\varepsilon ^{-1}\begin{bmatrix} d\beta _{\varPhi }\\ d\beta _{\varPsi } \end{bmatrix}. \end{aligned}$$

Note that the modes are pairwisely independent. We recall the notation in Def. 19-3 that $\lambda _k^s=\alpha _k^s+i\omega _k^s$. If we express ${\tilde{g}}_k(t)={\tilde{g}}_{k}^{Re}(t)+i{\tilde{g}}_{k}^{Im}(t),\;\forall k\in {\mathbb {Z}}{\setminus }\{0\}$, then we can find the solution for each pair of ${\tilde{g}}_{k}^{Re}(t)$ and ${\tilde{g}}_{k}^{Im}(t)$ explicitly.

1. For every $k\in \{2,3, \ldots \}$, ${\tilde{g}}_{\pm k}(t)={\tilde{g}}_{k}^{Re}(t)\pm i{\tilde{g}}_{k}^{Im}(t)$, and $[{\tilde{g}}_{k}^{Re}(t),{\tilde{g}}_{k}^{Im}(t)]^T$ are solved by
  5.2 $$\begin{aligned} \begin{aligned} \begin{bmatrix} {\tilde{g}}_k^{Re}\\ {\tilde{g}}_{k}^{Im} \end{bmatrix}(t)&=e^{\frac{\alpha _k^{s}(t-t_0)}{\varepsilon ^2}}\begin{bmatrix} cos\left( \frac{\omega _k^{s} (t-t_0)}{\varepsilon ^2}\right) &{} -sin\left( \frac{\omega _k^{s} (t-t_0)}{\varepsilon ^2}\right) \\ sin\left( \frac{\omega _k^{s} (t-t_0)}{\varepsilon ^2}\right) &{} cos\left( \frac{\omega _k^{s} (t-t_0)}{\varepsilon ^2}\right) \end{bmatrix}\begin{bmatrix} {\tilde{g}}_k^{Re}(0)\\ {\tilde{g}}_{k}^{Im}(0) \end{bmatrix}\\&\quad +\frac{\sqrt{q_k}e^{\frac{\alpha _k^{s}t}{\varepsilon ^2}}}{\varepsilon }\begin{bmatrix} \int _{t_0}^te^{-\frac{\alpha _k^{s}s}{\varepsilon ^2}}cos\left( \frac{\omega _k^{s} \sigma }{\varepsilon ^2}\right) d\beta _k(\sigma )-\int _{t_0}^te^{-\frac{\alpha _k^{s}\sigma }{\varepsilon ^2}}sin\left( \frac{\omega _k ^{s}\sigma }{\varepsilon ^2}\right) d\beta _{-k}(\sigma )\\ \int _{t_0}^te^{-\frac{\alpha _k^{s}\sigma }{\varepsilon ^2}}sin\left( \frac{\omega _k^{s} \sigma }{\varepsilon ^2}\right) d\beta _k(\sigma )+\int _{t_0}^te^{-\frac{\alpha _k^{s}\sigma }{\varepsilon ^2}}cos\left( \frac{\omega _k^{s} \sigma }{\varepsilon ^2}\right) d\beta _{-k}(\sigma ) \end{bmatrix} \end{aligned}\nonumber \\ \end{aligned}$$
  The stationary solution (as $t_0\rightarrow -\infty$) to (5.1a) and (5.1b) is given as $g_k^*=g_k^{Re^*}+ig_{k}^{Im^*}$, where $g_k^{Re^*}$ and $g_{k}^{Im^*}$ are independent Gaussian processes with
  $$\begin{aligned} {\mathbb {E}}[ g_k^{Re^*}(t)]={\mathbb {E}}[ g_k^{Im^*}(t)]=0 \end{aligned}$$
  and covariance matrix
  5.3 $$\begin{aligned} Cov(t,\sigma ) =\begin{bmatrix} {\mathbb {E}}[g_k^{Re^*}(t)g_k^{Re^*}(\sigma )] &{} {\mathbb {E}}[g_k^{Re^*}(t)g_k^{Im^*}(\sigma )]\\ {\mathbb {E}}[g_k^{Im^*}(t)g_k^{Re^*}(\sigma )] &{} {\mathbb {E}}[g_k^{Im^*}(t)g_k^{Im^*}(\sigma )] \end{bmatrix}=- \frac{q_k}{2\alpha _k^{s}}e^{\frac{\alpha _k^{s}|t-\sigma |}{\varepsilon ^2}}I_{2\times 2}. \nonumber \\ \end{aligned}$$
2. The solution to (5.1c) is given explicitly as,
  5.4 $$\begin{aligned} \begin{bmatrix} {\tilde{\varPhi }}_\delta (t)\\ {\tilde{\varPsi }}_\delta (t) \end{bmatrix}(t)= & {} e^{\frac{\alpha _{\gamma _1}^{s}(t-t_0)}{\varepsilon ^2}}P\begin{bmatrix} cos\left( \frac{\omega _{\gamma _1}^{s} (t-t_0)}{\varepsilon ^2}\right) &{} -sin\left( \frac{\omega _{\gamma _1}^{s} (t-t_0)}{\varepsilon ^2}\right) \\ sin\left( \frac{\omega _{\gamma _1}^{s} (t-t_0)}{\varepsilon ^2}\right) &{} cos\left( \frac{\omega _{\gamma _1}^{s} (t-t_0)}{\varepsilon ^2}\right) \end{bmatrix}P^{-1}\begin{bmatrix} {\tilde{\varPhi }}_\delta (0)\\ {\tilde{\varPsi }}_\delta (0) \end{bmatrix}\nonumber \\{} & {} +\varepsilon ^{-1}\int _{t_0}^te^{\frac{\alpha _{\gamma _1}^{s}(t-\sigma )}{\varepsilon ^2}}PR_{t,\sigma }P^{-1}\begin{bmatrix} d\beta _{\varPhi }(\sigma )\\ d\beta _{\varPsi }(\sigma ) \end{bmatrix} \nonumber \\ \end{aligned}$$
  where
  $$\begin{aligned} P=\begin{bmatrix} 0 &{} 1\\ Im(\nu _{\psi _1}) &{} Re(\nu _{\psi _1})\\ \end{bmatrix},\;\;R_{t,\sigma }=\begin{bmatrix} cos\left( \frac{\omega _{\gamma _1} ^{s}(t-\sigma )}{\varepsilon ^2}\right) &{} -sin\left( \frac{\omega _{\gamma _1}^{s} (t-\sigma )}{\varepsilon ^2}\right) \\ sin\left( \frac{\omega _{\gamma _1}^{s} (t-\sigma )}{\varepsilon ^2}\right) &{} cos\left( \frac{\omega _{\gamma _1} ^{s}(t-\sigma )}{\varepsilon ^2}\right) \end{bmatrix} \end{aligned}$$
  and $\nu _{\psi _1}$ is defined in Sect. A-5. Therefore, the stationary solution (as $t_0\rightarrow -\infty$) to (5.4) is given as
  $$\begin{aligned} {\mathbb {E}}[ \varPhi ^*_\delta (t)]={\mathbb {E}}[ \varPsi ^*_\delta (t)]=0 \end{aligned}$$
  and the covariance matrix
  5.5 $$\begin{aligned} Cov(t,\sigma ) =\varepsilon ^{-2}\int _0^{t\wedge \sigma } e^{\frac{\alpha _{\gamma _1}^{s}(t-r)}{\varepsilon ^2}}(PR_{t,r}P^{-1})(PR_{t,r}P^{-1})^T dr \end{aligned}$$

#### Remark 28

Note that the integral in (5.5) can be explicitly calculated. However, we use the implicit expression for the rest of the derivation.

### Evaluation of $\hat{z}(t)$

Since every operator in (4.5a), including *B*, $K^{-1}$, $L_s^{-1}$ and $\langle \nu ^*,\cdot \rangle _{U}$, is given explicitly, after some cumbersome calculation, we obtain

5.6 $$\begin{aligned} \hat{B}(\hat{x},L_s^{-1}P_sB(\hat{x},\hat{x})) =-\frac{{\mathcal {K}}_1{\mathcal {K}}_2}{4\lambda _2^s} \hat{z}^2{\overline{\hat{z}}}=- \frac{{\mathcal {K}}_1{\mathcal {K}}_2\lambda _{-2}^s\hat{z}^2{\overline{\hat{z}}}}{4(\alpha _2^{s2}+\omega _2^{s2})}=:h\lambda _{-2}^s\hat{z}^2\overline{\hat{z}}, \end{aligned}$$

where we have used notations defined in Def. 19-3. Similarly,

5.7 $$\begin{aligned} \hat{B}(\hat{x}, L_s^{-1}P_sB(y^*,y^*))= & {} N_1(\omega )\overline{\hat{z}}+N_2(\omega )\hat{z}-\frac{{\mathcal {K}}_1}{4l_c}\hat{z}^2\overline{\hat{z}}, \end{aligned}$$

5.8 $$\begin{aligned} \hat{B}(\hat{x}, L_s^{-1}P_sB(\hat{x},y^*))= & {} N_3(\omega )\overline{\hat{z}}^2, \end{aligned}$$

5.9 $$\begin{aligned} \hat{B}(P_cB(\hat{x},y^*),\;L_s^{-1}y^*)= & {} N_4(\omega )\hat{z}+N_5(\omega )\overline{\hat{z}}, \end{aligned}$$

5.10 $$\begin{aligned} \hat{B}(P_cB(y^*,y^*),\;L_s^{-1}y^*)= & {} N_6(\omega ). \end{aligned}$$

From Lemma 21,

5.11 $$\begin{aligned} \begin{aligned} \varepsilon ^{-1}\hat{B}(y^*,y^*)&=:N_7(\omega )\hat{z}+N_8(\omega )\overline{\hat{z}}+N_9(\omega )+N_{10}(\omega )\overline{\hat{z}}^2. \end{aligned} \end{aligned}$$

We also have

5.12 $$\begin{aligned} \begin{aligned} \hat{{{\textbf {F}}}}(\hat{x}+\hat{y})&=N_{11}(\omega )\overline{\hat{z}}^2+N_{12}(\omega )\hat{z}+N_{13}(\omega )\overline{\hat{z}}+N_{14}(\omega )-\frac{{\mathcal {K}}_1\psi _c'''}{2\psi _{c,\mu _c}''}\hat{z}^2\overline{\hat{z}}\end{aligned} \end{aligned}$$

For the stochastic term,

5.13 $$\begin{aligned} \hat{B}_1(\hat{x},L_s^{-1}P_sdW_t)=-\frac{{\mathcal {K}}_1}{2}\left[ \hat{z}(l_{11}d\beta _{\varPhi }+l_{12}d\beta _{\varPsi })+\frac{ \overline{\hat{z}}\sqrt{q_2}(d\beta _2+id\beta _{-2})}{\lambda _2^s}\right] \qquad \end{aligned}$$

The detailed information of the above shorthand notations $N_i(\omega )$ for $i\in \{1,2,\ldots ,14\}$ are given in Appendix D, where $\omega$ represents the randomness generated from the stable modes which are excited by noise terms. Making use of the results above (from Equation (5.6) to (5.13)), the solution of $\hat{z}$ can be determined by

5.14 $$\begin{aligned} \begin{aligned} \hat{z}(t)&= \hat{z}(0)+\int _0^t(\lambda _1(\mathfrak {q})+2N_2(\omega )+4N_4(\omega )+N_7(\omega )+N_{12}(\omega ))\hat{z}dt+ \int _0^t (2h\lambda _{-2}^s-j) \hat{z}^2\overline{\hat{z}}dt\\&\quad +\int _0^t(2N_1(\omega )+4N_5(\omega )+N_8(\omega )+N_{13}(\omega ))\overline{\hat{z}}dt+ \int _0^t(4N_3(\omega )+N_{10}(\omega )+N_{11}(\omega ))\overline{\hat{z}}^2 dt\\&\quad + \int _0^t(2N_6(\omega )\hat{z}+ N_9(\omega )+ N_{14}(\omega ))dt+2\int _0^tB_1(\hat{z},L_s^{-1}P_sdW_t) +{\mathcal {O}}(\varepsilon ), \\ \end{aligned}\nonumber \\ \end{aligned}$$

where $j:=\frac{{\mathcal {K}}_1\psi _c'''}{2\psi _{c,\mu _c}''}+\frac{{\mathcal {K}}_1}{2l_c}$, and *h* is defined in (5.6).

### Approximation of $\hat{z}(t)$

It is still not easy to evaluate (5.14). However, we observe that

5.15 $$\begin{aligned}{} & {} {\mathbb {E}}[N_i(t)]={\mathbb {E}}[N_i(0)]=0,\;i\in \{1,3,5,6,8,9,10,11,13,14\}, \end{aligned}$$

5.16 $$\begin{aligned}{} & {} {\mathbb {R}}\ni {\mathbb {E}}[N_2(t)]\ne 0 \;\;\text {and}\;\; {\mathbb {R}}\ni {\mathbb {E}}[N_{12}(t)]\ne 0, \end{aligned}$$

5.17 $$\begin{aligned}{} & {} {\mathbb {C}}\ni {\mathbb {E}}[N_4(t)]\ne 0\;\;\text {and}\;\; {\mathbb {C}}\ni {\mathbb {E}}[N_7(t)]\ne 0. \end{aligned}$$

Intuitively, we would like to replace $N_i$ with $\overline{N}_i:={\mathbb {E}}[N_i(0)]$ for $i\in \{1,\ldots ,14\}$. The solution (5.14) can still be approximated in some sense with small error (the estimation relies on (Blömker et al. (2007), Corollary 4.5)). We rephrase the statement of (Blömker et al. (2007), Corollary 4.5) and provide it in the following theorem.

#### Theorem 29

Let *f* be an $\tilde{\alpha }$-Hölder continuous function on $[0,\tau ^*]$. Assume that for every $\varepsilon >0$ and fixed $\kappa >0$, there exist a constant $C_1$ such that

$$\begin{aligned} {\mathbb {E}}\left[ \left\| \int _s^t(N(r)-\overline{N}(r)dr\right\| _\alpha ^p\right] \le C_1(t-s)^{p/2}\varepsilon ^p. \end{aligned}$$

Then, for every $\gamma <2\tilde{\alpha }/(1+2\tilde{\alpha })$, there exists a constant *C* depending only on p and $\gamma$ such that

$$\begin{aligned} {\mathbb {E}}\left[ \sup \limits _{t\in [0,\tau ^*]}\left| \int _0^t f(s)(N(s)-\overline{N}(s))ds\right| ^p\right] \le C\varepsilon ^{\gamma p}\left( {\mathbb {E}}\left[ \Vert f\Vert _{C^{\tilde{\alpha }}}\right] ^{2p}\right) ^{1/2}, \end{aligned}$$

where $\Vert \cdot \Vert _{C^{\tilde{\alpha }}}$ denotes the $\tilde{\alpha }$-Hölder norm.

#### Remark 30

The above theorem can be used to approximate $\hat{z}(t)$ by replacing $N_i$ with $\overline{N}_i$ for each $i\in \{1,2,\ldots ,14\}$, and the error is within ${\mathcal {O}}(\varepsilon ^{p\gamma })$ in $p^{\text {th}}$-moment. In (5.14), $f_1=f_5=f_8=f_{13}=\overline{\hat{z}}$, $f_2=f_4=f_7=f_{12}=\hat{z}$, $f_3=f_{10}=f_{11}=\overline{\hat{z}}^2$, $f_6=f_9=f_{14}=1$. Note that for $\tilde{\alpha }<1/2$, we have $f_i$’s satisfy the condition in Theorem 29, and as a concequence we can choose $\gamma <1/2$. To use Theorem 29, it suffices to show the condition ${\mathbb {E}}\left[ \left\| \int _s^t(N_i-\overline{N}_i)dr\right\| _\alpha ^p\right] \le C_1(t-s)^{p/2}\varepsilon ^p$ holds. We only show the cases when $k\in {\mathbb {Z}}\setminus \{0\}$ (the case for $[{\hat{\varPhi }}_\delta ^*,{\hat{\varPsi }}_\delta ^*]^T$ is similar).

Let $g_k^\circ$ represent either $g_k^{Re^*}$ or $g_{k}^{Im^*}$ (from (5.2)), we have the following estimations.

#### Lemma 31

For every $k\in {\mathbb {Z}}\setminus \{0\}$, we have

$$\begin{aligned} {\mathbb {E}}\left[ \left( \int _\sigma ^t g^\circ _k(r)dr\right) ^{2p}\right] \le \frac{q_k^p\varepsilon ^{2p}}{(\alpha _k^{s})^{2p}} \varepsilon ^{2p} (t-\sigma )^p \end{aligned}$$

#### Proof

Let $$p=1$$, then

$$\begin{aligned} \begin{aligned} {\mathbb {E}}\left[ \left( \int _\sigma ^t g^\circ _k(r)dr\right) ^2\right]&= {\mathbb {E}}\left[ \left( \int _\sigma ^t g^\circ _k(r)dr\right) \left( \int _\sigma ^t g^\circ _k(u)du\right) \right] \\&=\int _\sigma ^t\int _\sigma ^t{\mathbb {E}}[g^\circ _k(r)g^\circ _k(u)]drdu\\&= -2\int _\sigma ^t\int _\sigma ^t \frac{q_k}{2\alpha _k^{s}}e^{\frac{\alpha _k^{s}(r-u)}{\varepsilon ^2}}drdu\\&= \frac{q_k\varepsilon ^2}{(\alpha _k^{s})^2}\left( t-\sigma -\frac{ \varepsilon ^2}{(\alpha _k^{s})^2}(1-e^{\frac{\alpha _k^{\varepsilon } (t-\sigma )}{\varepsilon ^2}})\right) \le \frac{q_k\varepsilon ^2}{(\alpha _k^{s})^2}(t-\sigma ), \end{aligned} \end{aligned}$$

where the 2nd equality is by Fubini. Let $I_k:=\int _\sigma ^t g^\circ _k(r)dr$, then $I_k$ is Gaussian with ${\mathbb {E}}[I_k]=0$ and ${\mathbb {E}}[I_k^2]\le - \frac{q_k\varepsilon ^2}{\alpha _k^{s2}}(t-\sigma )$. Therefore,

$$\begin{aligned} {\mathbb {E}}[|I_k|^{2p}]={\mathbb {E}}[I_k^2]^p\le \left( \frac{q_k\varepsilon ^2}{(\alpha _k^{s})^2}(t-\sigma )\right) ^p \end{aligned}$$

for every $$p>0$$.

#### Lemma 32

For every $k\in {\mathbb {Z}}{\setminus }\{0,\pm 1\}$, $k\ne l$ and $k+l\ne 0$, there exists a constant $C>0$ such that

$$\begin{aligned} {\mathbb {E}}\left[ \left( \int _\sigma ^t g^\circ _k(r)g^\circ _l(r)dr\right) ^{2p}\right] \le C\left( \frac{q_kq_l}{\alpha _k^s\alpha _l^s}\right) ^p(t-\sigma )^{p}\varepsilon ^{2p} \end{aligned}$$

#### Lemma 33

For every $k\in {\mathbb {Z}}\setminus \{0,\pm 1\}$, $k=l$ or $k+l= 0$, there exists a constant $C>0$ such that

$$\begin{aligned} {\mathbb {E}}\left[ \left( \int _\sigma ^t g^\circ _k(s)g^\circ _l(s)-{\mathbb {E}}[g^\circ _k(s)g^\circ _l(s)] ds\right) ^{2p}\right] \le C\left( \frac{q_kq_l}{\alpha _k^s\alpha _l^s}\right) ^p(t-\sigma )^{p}\varepsilon ^{2p} \end{aligned}$$

#### Lemma 34

For every $k\in {\mathbb {Z}}\setminus \{0,\pm 1\}$, there exists a constant $C>0$ such that

$$\begin{aligned} {\mathbb {E}}\left[ \left( \int _\sigma ^t g^\circ _k(s)g^\circ _l(s)g^\circ _j(s)ds\right) ^{2p}\right] \le C\left( \frac{q_kq_lq_j}{\alpha _k^s\alpha _l^s\alpha _j^s}\right) ^p(t-\sigma )^{p}\varepsilon ^{2p} \end{aligned}$$

The proof for Lemma 32 to 34 is based on expanding the product of integrals that have Gaussian properties. The idea follows the proof of (Blömker et al. (2007), Lemma 4.1). We do not provide the proof in this paper as we can simply treat the complex-valued $g_k^*$ as we did in Lemma 31, and the rest follows exactly as (Blömker et al. (2007), Lemma 4.1).

#### Corollary 35

For every $i\in \{1,2,\ldots ,14\}$, there exists a constant $C>0$ such that ${\mathbb {E}}\left[ \left\| \int _s^t(N_i-\overline{N}_i)dr\right\| _\alpha ^p\right] \le C(t-s)^{p/2}\varepsilon ^p$.

#### Proof

By Def. 6 and Assumption 15, combining the definition of $N_i$ and $\overline{N}_i$, it can be shown that the bounds generated from Lemma 32 to 34 converge. $\square$

Renaming some constant quantities, we put (5.14) in a concise form. To this end, let

$$\begin{aligned} c_1+ic_2:={\mathbb {E}}[2N_2+4N_4+N_7+N_{12}]. \end{aligned}$$

We also define

$$\begin{aligned} \begin{aligned}&\sigma _1:=-\frac{{\mathcal {K}}_1}{2} l_{11},\;\;\sigma _2:=-\frac{{\mathcal {K}}_1}{2}l_{12}\\&\sigma _3:=-\frac{{\mathcal {K}}_1\alpha _2^s\sqrt{q_2}}{2((\alpha _2^{s})^2+(\omega _2^{s})^2)},\;\;\sigma _4:=-\frac{{\mathcal {K}}_1\omega _2^s\sqrt{q_2}}{2((\alpha _2^{s})^2+(\omega _2^{s})^2)}\\ \end{aligned} \end{aligned}$$

and

$$\begin{aligned} M(v^a)=\begin{bmatrix} \sigma _1v_1^a &{} \sigma _2v_1^a &{} \sigma _3v_1^a-\sigma _4v_2^a &{} \sigma _4v_1^a+\sigma _3v_2^a\\ \sigma _1v_2^a &{} \sigma _2v_2 ^a&{} -\sigma _3v_2^a-\sigma _4v_1^a &{} -\sigma _4v_2^a+\sigma _3v_1^a\\ \end{bmatrix}_{2\times 4}. \end{aligned}$$

Now we use $\hat{z}=x_1+ix_2$, let $v^a:=[x_1,x_2]^T$ represent the converted amplitudes. Moreover, we set

5.18 $$\begin{aligned}{} & {} {\mathfrak {A}}(\mathfrak {q}):=\begin{bmatrix} \alpha _c(\mathfrak {q})+c_1 &{} -\omega _c(\mathfrak {q})-c_2\\ \omega _c(\mathfrak {q})+c_2 &{}\alpha _c(\mathfrak {q})+c_1 \end{bmatrix}_{2\times 2}, \end{aligned}$$

5.19 $$\begin{aligned}{} & {} {\mathfrak {B}}:=\begin{bmatrix} 2h\alpha _2^s -j &{} 2h\omega _2^s\\ -2h\omega _2^s &{} 2h\alpha _2^s -j \end{bmatrix}_{2\times 2}, \end{aligned}$$

5.20 $$\begin{aligned}{} & {} {\mathcal {W}}=[\beta _{\varPhi },\beta _{\varPsi },\beta _{2},\beta _{-2}]^{\text {T}}, \end{aligned}$$

where $\alpha _c,\omega _c$ are defined in Def. 19. Then (5.14) is equivalent to

5.21 $$\begin{aligned} \begin{aligned}&v^a(t)=v^a(0)+\int _0^t{\mathfrak {A}}(\mathfrak {q}) v^adt+\int _0^t|v^a|^2{\mathfrak {B}}v^adt+\int _0^tM(v^a)dW_s+{\text {Er}}(t)\\&{\mathbb {E}}\left[ \sup _{t\in [0,\tau ^*]}\Vert {\text {Er}}(t)\Vert ^p\right] ={\mathcal {O}}(\varepsilon ^{p/2-})\\&v^a(0)=[Re(\hat{z}(0)),Im(\hat{z}(0)]^T \end{aligned} \end{aligned}$$

where ${\text {Er}}$ is an error term that vanishes as $\varepsilon \rightarrow 0$.

#### Remark 36

$v^a$ from (5.21) is the finite-dimensional (2d) representation of the original SPDE (3.1) close to the stall bifurcation point. However, the small error term ${\text {Er}}(t)$ implicitly contains stochastic components from the stable modes. Below we use the Martingale problem (Ethier and Kurtz 2009; Stroock and Varadhan 2007; Sviridenko 1990) to derive a self-contained Markov process approximation for $v^a$.

## Weak Convergence of the Probability Measure

In this section, we investigate how the stopped solution to (5.21) or the probability measure converge as $\varepsilon \rightarrow 0$. Given the probability law $\nu ^\varepsilon$ of the stopped process $\{{\hat{v}}(t\wedge \tau ^*)\}_{t\ge 0}$ driven by noise with intensity $\varepsilon$, the process $\{v^a(t\wedge \tau ^*)\}_{t\ge 0}$ of (5.21) lies in the induced canonical space with probability law $\nu _c^{\varepsilon }=P_c\nu ^{\varepsilon }:=\nu ^\varepsilon \circ P_c^{-1}$. Here we show that the unique limit $\nu _c$ of $\nu _c^\varepsilon$ solves the Martingale problem (Ethier and Kurtz 2009) related to the 2-dimensional SDE for $t\in [0,T]$:

6.1 $$\begin{aligned} {\tilde{v}}^a={\tilde{v}}^a(0)+\int _0^t{\mathfrak {A}}(\mathfrak {q}){\tilde{v}}^adt+\int _0^t|{\tilde{v}}^a|^2{\mathfrak {B}}{\tilde{v}}^adt+\int _0^t\varSigma ({\tilde{v}}^a) d\beta _t \end{aligned}$$

where ${\tilde{v}}^a=[{\tilde{v}}^a_1,{\tilde{v}}^a_2]^T$, $\beta$ stands for a two-dimensional standard Wiener process, and

6.2 $$\begin{aligned} \varSigma ({\tilde{v}}^a):= \begin{bmatrix} \left( \sum \limits _{i=1}^4\sigma _i\right) {\tilde{v}}^a_1+\left( \sigma _3-\sigma _4\right) {\tilde{v}}^a_2\\ \left( \sum \limits _{i=1}^2\sigma _i-\sum \limits _{i=3}^4\sigma _i\right) {\tilde{v}}^a_2+\left( \sigma _3-\sigma _4\right) {\tilde{v}}^a_1 \end{bmatrix} \end{aligned}$$

### Theorem 37

Suppose $2h\alpha _2^s-j<0$ in (5.19). For each fixed $T>0$, the sequence of measures $\nu _c^{\varepsilon }$ converges weakly to $\nu _c$, which is the law of the solution ${\tilde{v}}^a\in C([0,T];\mathbb {R}^2)$ to (6.1).

To prove the above theorem, we need to demonstrate that: (1) the family of probability measure $\{\nu ^{\varepsilon }\}$ or $\{\nu _c^{\varepsilon }\}$ is tight, such that there exists a weakly convergent subsequence within that family, and (2) every accumulation point of $\nu _c^{\varepsilon }$ is the unique solution to the Martingale problem associated with (6.1).

### Tightness of $\{\nu _c^{\varepsilon }\}$

The proof falls in standard procedures. Let $f(\cdot )=\Vert \cdot \Vert ^p$, and $h=v^a-{\text {Er}}$. Then, by (Blömker and Fu (2020), Lemma 4.9), we have

6.3 $$\begin{aligned}{} & {} {\text {Tr}}[f''(h(\sigma )){\textbf{M}}(h(\sigma )+{\text {Er}}(\sigma )){\textbf{M}}^*(h(\sigma )+{\text {Er}}(\sigma ))]\le Cp(p-1)\Vert h(\sigma )\Vert ^{p-2}\Vert h(\sigma )\nonumber \\{} & {} \quad +{\text {Er}}(\sigma )\Vert ^2. \end{aligned}$$

Applying Itô formula to $\Vert h\Vert ^p$ for $p\ge 2$ and use the above inequality, for all $t\in [0,T]$, we have

6.4 $$\begin{aligned} \begin{aligned}&\Vert h(t\wedge \tau ^*)\Vert ^p-\Vert h(0)\Vert ^p\\&\quad \le p\int _0^{t\wedge \tau ^*} \Vert h(s)\Vert ^{p-2}\langle \mathfrak {A}(\mathfrak {q})(h(s)+{\text {Er}}(s)), h(s)+{\text {Er}}(s)\rangle ds\\&\qquad +\int _0^{t\wedge \tau ^*}\Vert h(s)\Vert ^{p-2}\langle |h(s)+{\text {Er}}(s)|^2\mathfrak {B}|h(s)+{\text {Er}}(s)|, h(s)+{\text {Er}}(s)\rangle ds\\&\qquad +Cp(p-1)\int _0^{t\wedge \tau ^*} \Vert h(s)\Vert ^{p-2}\Vert h(s)+{\text {Er}}(s)\Vert ^2 ds\\&\qquad +\int _0^{t\wedge \tau ^*}\Vert h(s)\Vert ^{p-2}\langle h(s),M(h(s)+{\text {Er}}(s))d{\mathcal {W}}_s\rangle . \end{aligned} \end{aligned}$$

By the assumption, we can verify that there exists some $b<0$ such that $\langle x, |x|^2{\mathfrak {B}}x\rangle \le b|x|^4$ for all $x\in {\mathbb {R}}^2$. Consequently, the first three terms can be bounded by

6.5 $$\begin{aligned} C\int _0^{t\wedge \tau ^*}\Vert h(s)\Vert ^{p-2}\Vert h(s)+{\text {Er}}(s)\Vert ^2ds. \end{aligned}$$

By triangle inequality and Young’s inequality (for products), (6.5) can be further bounded by

6.6 $$\begin{aligned} C \int _0^{t\wedge \tau ^*}\Vert h(s)\Vert ^{p}ds+C\int _0^{t\wedge \tau ^*}\Vert {\text {Er}}(s)\Vert ^{p}ds. \end{aligned}$$

Applying Burkholder–Davis–Gundy inequality and then Young’s inequality to the last term in (6.4), we can obtain the bound

6.7 $$\begin{aligned} \begin{aligned}&\mathbb {E}^{\nu _c^\varepsilon }\sup _{0\le t\le \tau ^*} \Vert h(s)\Vert ^{p-2}\langle h(s),M(h(s)+{\text {Er}}(s))d{\mathcal {W}}_s\rangle \\&\quad \le C\int _0^{t\wedge \tau ^*}\mathbb {E}^{\nu _c^\varepsilon } \sup _{0\le s\le \tau ^*} \Vert h(s)\Vert ^pds+C. \end{aligned} \end{aligned}$$

Combining the above, we have

$$\begin{aligned} \mathbb {E}^{\nu _c^\varepsilon }\sup _{0\le t\le \tau ^*}\Vert h(t)\Vert ^p\le C\int _0^{t\wedge \tau ^*}\mathbb {E}^{\nu _c^\varepsilon } \sup _{0\le s\le \tau ^*} \Vert h(s)\Vert ^pds+C. \end{aligned}$$

By Gronwall’s inequality, we can verify that $\mathbb {E}^{\nu _c^\varepsilon }\sup _{0\le t\le \tau ^*}\Vert h(t)\Vert ^p\le C$, which implies the uniform boundedness of the quantity $\mathbb {E}^{\nu _c^\varepsilon }\sup _{0\le t\le \tau ^*}\Vert v^a(t)\Vert ^p$. The uniform tightness of $\{\nu _c^\varepsilon \}$ follows.

#### Remark 38

Note that by introducing the compact operator $G_{\alpha }: L^p([0,T]; U))\rightarrow C([0,T]; U)$ for $0<1/p< \alpha \le 1$ and $t\in [0,T]$:

$$\begin{aligned} G_{\alpha }f(t)=\int _0^t (t-s)^{\alpha -1}S(t-s)f(s)ds,\;\;f\in L^p([0,T], H), \end{aligned}$$

as well as $Y_{\alpha }^{\varepsilon }(t)=\varepsilon \int _0^t (t-r)^{-\alpha }S(t-r)dW(r),$ the mild solution can be expressed as

6.8 $$\begin{aligned} v(t)=S(t)v_0+G_1(\varepsilon ^{-1}B(v,v)+F(v))(t)+\frac{\sin \alpha \pi }{\pi }G_{\alpha }(Y_{\alpha }^{\varepsilon })(t). \end{aligned}$$

The compactness of $G_{\alpha }$ has been shown in (Da Prato and Zabczyk (2014), Proposition 8.4). To show the tightness of $\{\nu ^\varepsilon \}$, it suffices to show that for each $\eta >0$, there exist uniformly bounded sets $J_\eta$ and $H_\eta$ of $\varepsilon ^{-1}B(v,v)+{\textbf{F}}(v)$ and $Y_\alpha ^\varepsilon$, respectively, as $L^p([0,T],U)$ functions, such that $\nu ^\varepsilon (K_\eta )\ge 1-\eta$ for $K_\eta =\{S(t)v_0+G_1(J_\eta )+\frac{\sin \alpha \pi }{\pi }G_\alpha (H_\eta )\}$. However, for a fixed $p\ge 2$ we can only find $C(\varepsilon )>0$ for each $\varepsilon >0$ such that

6.9 $$\begin{aligned} \mathbb {E}^\varepsilon \left[ \int _0^{t\wedge \tau ^*} |Y_{\alpha }^{\varepsilon }(s)|^pds\right] \le C(\varepsilon ),\;\;\forall t\in [0,T], \end{aligned}$$

and

6.10 $$\begin{aligned} \mathbb {E}^\varepsilon \left[ \int _0^{t\wedge \tau ^*} |\varepsilon ^{-1}B+{\textbf{F}}|^pds\right] \le C(\varepsilon )\;\;\forall t\in [0,T]. \end{aligned}$$

The nonuniform bounds fail to guarantee the tightness of $\{\nu ^\varepsilon \}$.

#### Proposition 39

Suppose $2h\alpha _2^s-j<0$ in (5.19). Then, for $\hat{\mu }\in {\mathscr {B}}(\mu _c,\varepsilon ^2{\mathfrak {q}})$, we have ${\mathbb {P}}[\tau ^*<T]\rightarrow 0$ as $\varepsilon \rightarrow 0$ for all $T>0$.

#### Proof

By the same procedure as in (Blömker et al. (2007), Corollary 3.7), one can show that, for each *T*,

6.11 $$\begin{aligned} {\mathbb {P}}[\tau ^*<T]={\mathbb {P}}\left[ \sup _{0\le t\le T}\Vert \hat{x}(t)+\hat{y}(t)\Vert _\alpha \ge \varepsilon ^{-\kappa }\right] \le {\mathbb {P}}[K|v^a(\tau ^*)|\ge \varepsilon ^{-\kappa }] +C\varepsilon ^p,\nonumber \\ \end{aligned}$$

where the small term $C\varepsilon ^p$ is contributed by the stable mode. By the assumption and the uniform boundedness of $\mathbb {E}^{\nu _c^\varepsilon }\sup _{0\le t\le \tau ^*}\Vert v^a(t)\Vert ^p$ for each $p\ge 2$ from the above proof, the conclusion follows immediately by (6.11) and Markov inequality.

#### Remark 40

Note that we always have $h>0$ by definition, which implies that $h\alpha _2^s<0$. On the other hand, the term *j* in (5.19) is generated as the result of homogenization. The configuration parameters of jet engine compressors should be carefully designed to guarantee the satisfaction of the condition. The intuitive purpose of introducing such a condition is to guarantee that the cubic nonlinearity of the homogenized system still possesses certain level dissipativity.

### Martingale Problem

Given a test function $\phi \in C_0^{\infty }(P_cU)$, for each $\mathfrak {q}$, the generator ${\mathcal {A}}(\mathfrak {q})$ of (6.1) is given by

6.12 $$\begin{aligned} {\mathcal {A}}(\mathfrak {q})\phi (x)=\langle {\mathfrak {A}}(\mathfrak {q})x+|x|^2{\mathfrak {B}}x,\; \nabla \phi \rangle +\frac{1}{2}\sum \limits _{i,j}^2\left( \varSigma \varSigma ^T\right) _{ij}\frac{\partial ^2\phi }{\partial x_i\partial x_j}. \end{aligned}$$

Then, by defining

6.13 $$\begin{aligned} M_{t}^{\varepsilon }:=\phi (v^a-{\text {Er}})(t\wedge \tau ^*)-\phi (v^a)(0)-\int _0^{t\wedge \tau ^*}{\mathcal {A}}(\mathfrak {q})\phi (v^a-{\text {Er}})(s)ds, \end{aligned}$$

it is clear that $\{M^{\varepsilon }\}$ is a family of stopped martingales. Due to the boundedness of ${\text {Er}}$ and the smoothness of the test function $\phi$, there exists a process ${\tilde{{\text {Er}}}}(t)$ such that

6.14 $$\begin{aligned} M_{t}^{\varepsilon }=\phi (v^a)(t\wedge \tau ^*)-\phi (v^a)(0)-\int _0^{t\wedge \tau ^*}{\mathcal {A}}(\mathfrak {q})\phi (v^a)(s)ds+{\tilde{{\text {Er}}}}(t\wedge \tau ^*),\qquad \end{aligned}$$

and $\lim _{\varepsilon \rightarrow 0}{\mathbb {E}}^{\nu _c^{\varepsilon }}[\sup _{t\in [0,\tau ^*]}{\tilde{{\text {Er}}}}(t)]=0$, where ${\mathbb {E}}^{\nu _c^{\varepsilon }}$ is the expectation operator w.r.t. the measure $\nu _c^{\varepsilon }$. Therefore, for any $0\le r_1<r_2< \cdots<r_n\le s<t$ and $\{\psi _j;\;j=1,2,\ldots ,n\}\subset C(P_cU)$, we alternatively have

6.15 $$\begin{aligned} {\mathbb {E}}^{\nu _c^\varepsilon }\left[ \{M_t^{\varepsilon }-M_s^{\varepsilon }\}\prod \limits _{j=1}^n\varphi _j(v^a_{r_j})\right] =0 \end{aligned}$$

We also define the Martingale process w.r.t. (6.1) as

6.16 $$\begin{aligned} M_t=\phi (v^a_{t})-\phi (v^a_0)-\int _0^{t}{\mathcal {A}}(\mathfrak {q})\phi (v^a_s)ds. \end{aligned}$$

Since the smooth test function has a compact support, we can also justify that $\{M_{t\wedge \tau ^*}\}_{t\in [0,T]}$ is uniformly integrable. By the tightness of $\{\nu _c^{\varepsilon }\}$ on $P_cU$, we can find a convergent subsequence $\nu _c^{\varepsilon _n} \rightharpoonup \nu _c$ as $n\rightarrow \infty$ (where $\varepsilon _n\rightarrow 0$). Therefore, by Proposition 39 and the convergence of $\mathbb {E}^{\nu _c^{\varepsilon _n}}[\sup _{t\in [0,\tau ^*]}{\tilde{{\text {Er}}}}(t)]$, we have

6.17 $$\begin{aligned}{} & {} {\mathbb {E}}^{\nu _c}\left[ \{M_t-M_s\}\prod \limits _{j=1}^n\varphi _j(v^a_{r_j})\right] =\lim \limits _{n\rightarrow \infty } {\mathbb {E}}^{\nu _c^{\varepsilon _n}}\left[ \{M_{t\wedge \tau ^*}-M_{s\wedge \tau ^*}\}\prod \limits _{j=1}^n\varphi _j(v^a_{r_j})\right] \nonumber \\{} & {} =\lim \limits _{n\rightarrow \infty } {\mathbb {E}}^{\nu _c^{\varepsilon _n}}\left[ \{M_t^{\varepsilon _n}-M_s^{\varepsilon _n}\}\prod \limits _{j=1}^n\varphi _j(v^a_{r_j})\right] =0 \end{aligned}$$

which means every limit of $\nu _c^{\varepsilon _n}$ solves the martingale problem w.r.t. (6.16). Note that under the dissipativity and local Lipschitz continuity, by Yamada-Watanabe, the solution to the Martingale problem is unique, which means every limit point $\nu _c$ is unique, and therefor the claim in Theorem 37 holds.

#### Remark 41

Theorem 37 also implies that $v^{a}$ converges to ${\tilde{v}}^a$ in law.

## Conclusions

Based on recent advances in stochastic PDEs given in Blömker et al. (2007), this paper further develops the bifurcation analysis of the stochastic version of the Moore and Greitzer PDE model (1.4) for an axial flow compressor, in the presence of a Hopf bifurcation. Close to bifurcation, the null-space being finite-dimensional simplifies the analysis of such PDEs. We provides approximations for the state *g*(*t*) for the stall case in the neighborhood of the deterministic bifurcation point. The evolution equation for slow-varying coordinates ${\tilde{v}}^a$ is derived by a careful analysis of the coupling of slow-fast modes arising from the spectral gap.

As explained previously, in addition to the direct influence that the additive noise has on the critical modes, which we assumed to be identically zero, the additive stochastic components in the stable, heavily damped modes also contribute to the critical modes. These contributions enter the critical modes as multiplicative noise through the terms $N_i'={\mathbb {E}}[N_i(t)]$ for $i\in \{1,\ldots ,14\}$ in (5.14) and are eventually incorporated into the 2-dimensional SDE (5.21). Hence, the stochastic bifurcation points for stall are shifted due to the evolution (stochastic) of heavily damped modes, $\varPhi _\delta$ and $g_{\pm n}$ for all $n\in {\mathbb {Z}}^+$. As the intensity $\varepsilon \rightarrow 0$, we justified a weak convergence of the probability measure of the slow-varying processes. The approximated slow processes also converge in law to the solution to (6.1).

## A Properties of the Linear Operator

For references, we list crucial properties of the linear operator $L({{\hat{\mathfrak {q}}}})$.

1. $L({{\hat{\mathfrak {q}}}})$ generates an analytic compact $C_0$ semigroup ${\hat{S}}(t):=e^{L({{\hat{\mathfrak {q}}}})t}$ on *U* (Xiao 2008).
2. For each ${{\hat{\mathfrak {q}}}}$, there exist constants $\omega \ge 0$ and $M\ge 1$ such that
  $$\begin{aligned} \Vert {\hat{S}}(t)\Vert _U\le M e^{\omega t},\;\forall t>0. \end{aligned}$$
  For the stable projection, there exists a $\omega >0$ and $M>0$ such that
  $$\begin{aligned} \Vert P_s{\hat{S}}(t)\Vert _U\le M e^{-\omega t},\;\forall t>0. \end{aligned}$$
3. $L({\hat{\mathfrak {q}}})$ can be represented as
  A.1 $$\begin{aligned} L({\hat{\mathfrak {q}}})=\begin{bmatrix} L|_{{\mathcal {H}}}({\hat{\mathfrak {q}}}) &{} 0\\ 0 &{} L|_{{\mathbb {R}}^2}({\hat{\mathfrak {q}}}) \end{bmatrix}, \end{aligned}$$
  where $L|_{{\mathcal {H}}}: {\mathcal {H}}\rightarrow {\mathcal {H}}$ is the restriction of *L* onto ${\mathcal {H}}$, whilst *L* restricted to ${\mathbb {R}}^2$ is a $2\times 2$ matrix $L|_{{\mathbb {R}}^2}$. The decoupling of the eigenspace makes the flow of *g* and $(\varPhi ,\varPsi )$ invariant respectively under the semigroups $e^{L|_{{\mathcal {H}}}({\hat{\mathfrak {q}}})t}$ and $e^{L|_{{\mathbb {R}}^2}({\hat{\mathfrak {q}}})t}$.
4. We can expect the solution *v* to be in the domain of $L({\hat{\mathfrak {q}}})$ (a subspace of *U*), which is
  $$\begin{aligned} {\mathcal {D}}(L({\hat{\mathfrak {q}}}))=H^2_{0}\times {\mathbb {R}}\times {\mathbb {R}}. \end{aligned}$$
5. We verify the type of Hopf bifurcation by the sign of the indicator (Xiao 2008)
  A.2 $$\begin{aligned} \varDelta :=\frac{\psi _{c_0}+\iota \left[ 1+\frac{3}{2}\sqrt{1-\frac{\nu \varTheta }{3a\iota }}-\frac{1}{2}\left( \sqrt{1-\frac{\nu \varTheta }{3a\iota }}\right) ^3\right] }{\varTheta \left( 1+\sqrt{1-\frac{\nu \varTheta }{3a\iota }}\right) }-\frac{a}{4B^2\nu }. \end{aligned}$$
  In particular, the stall bifurcation happens when $\varDelta <0$.

## B Proof of Lemma 10

### Proof

The proof easily follows (Lord et al. (2014), Proposition 1.93). Initially, we have

$$\begin{aligned} {\mathcal {D}}(L|_{{\mathcal {H}}})=H^2_{0}(D)\subset U. \end{aligned}$$

For $u\in H^r$, we can write $u=\sum _{n\in {\mathbb {Z}}{\setminus }\{0\}} u_ne^{in\theta }$, then the Sobolev norm can be expanded as

$$\begin{aligned} \Vert u\Vert _{H^r}^2=\sum _{n\in {\mathbb {Z}}\setminus \{0\}} (1+n^2+ \cdots +n^{2r})|u_n|^2 \end{aligned}$$

However, as the discrete spectrum of $L|_{{\mathcal {H}}}$ are $\lambda _{n}\in {\mathbb {C}}\;(\forall n\in {\mathbb {Z}}\cap {\mathcal {I}})$, we can explicitly express $\Vert u\Vert _{r/2}^2$ by

$$\begin{aligned} \Vert u\Vert _{r/2}^2=\langle L^{r/2}u,KL^{r/2}u\rangle _{{\mathcal {H}}}=K\sum (\lambda _n\lambda _{-n})^{r/2}|u_n|^2=\sum \left( 1+\frac{am}{|n|}\right) (\lambda _n\lambda _{-n})^{r/2}|u_n|^2 \end{aligned}$$

By the definition of *K* and $\lambda _{n}$, we can find $C_1,C_2>0$ such that $C_1(1+n^2)^r\le \left( 1+\frac{am}{|n|}\right) (\lambda _n\lambda _{-n})^{r/2}\le C_2(1+n^2)^r$. We also have

$$\begin{aligned} \frac{1}{2^r}(1+n^2)^r\le (1+n^2+ \cdots +n^{2r})\le (1+n^2)^r \end{aligned}$$

Combine the above two sets of inequalities,

$$\begin{aligned} \frac{1}{C_2 2^r}\left( 1+\frac{am}{|n|}\right) (\lambda _n\lambda _{-n})^{r/2}\le (1+n^2+ \cdots +n^{2r})\le \frac{1}{C_1}\left( 1+\frac{am}{|n|}\right) (\lambda _n\lambda _{-n})^{r/2} \end{aligned}$$

Then, by the definition of the two norms, it is not hard to see that $\Vert u\Vert _{r/2}^2\sim \Vert u\Vert _{H^r}^2$. $\square$

## C Proof of Lemma 21

### Proof

Note that $\hat{B}(\hat{y},\hat{y})=\frac{a(\psi _c'')}{1+am}\sum _{k\in \{-2,-3, \ldots \}}^{k+l=1}\hat{g_k}\hat{g_l}$. From (4.2b) we keep the terms up to ${\mathcal {O}}(\varepsilon ^{-1})$ and regard the rest as higher order terms (h.o.t.), then

$$\begin{aligned} \begin{aligned} d\hat{g_l}&=\varepsilon ^{-2}\lambda _l^s\hat{g_l}dt+\varepsilon ^{-1}\langle \nu _l^*,P_sB(\hat{x}+\hat{y},\hat{x}+\hat{y})\rangle _{U}dt\\&\quad +\varepsilon ^{-1}\sqrt{q_l} (d\beta _l(t)+id\beta _{-l}(t))+\text {h.o.t.},\;\forall l\in \{3,4 \ldots \}, \end{aligned} \end{aligned}$$

and

$$\begin{aligned} \begin{aligned} d\hat{g_k}&=\varepsilon ^{-2}\lambda _k^s\hat{g_k}dt+\varepsilon ^{-1}\langle \nu _k^*,P_sB(\hat{x}+\hat{y},\hat{x}+\hat{y})\rangle _{U}dt\\&\quad +\varepsilon ^{-1}\sqrt{q_k}(d\beta _{-k}(t)-id\beta _{k}(t))+\text {h.o.t.}, \forall k\in \{-2,-3 \ldots \} \end{aligned} \end{aligned}$$

For $l\in \{3,4, \ldots \}$ we have,

$$\begin{aligned} \begin{aligned} \langle \zeta _l^*,P_sB(\hat{x}+\hat{y},\hat{x}+\hat{y})\rangle _{U}&=\langle \zeta _l^*,B(\hat{x},\hat{x})\rangle _{U}+2\langle \zeta _l^*,P_sB(\hat{x},\hat{y})\rangle _{U}+\langle \zeta _l^*,P_sB(\hat{y},\hat{y})\rangle _{U}\\&=0+ \left( \hat{z}{\mathcal {K}}_{l-1}{\hat{g}}_{l-1}+\overline{\hat{z}}{\mathcal {K}}_{l+1}{\hat{g}}_{l+1} \right) +{\mathcal {G}}_l \end{aligned} \end{aligned}$$

for $$k=-2$$,

$$\begin{aligned} \begin{aligned} \langle \zeta _k^*,P_sB(\hat{x}+\hat{y},\hat{x}+\hat{y})\rangle _{U}&=\langle \zeta _k^*,B(\hat{x},\hat{x})\rangle _{U}+2\langle \zeta _k^*,P_sB(\hat{x},\hat{y})\rangle _{U}+\langle \zeta _k^*,P_sB(\hat{y},\hat{y})\rangle _{U}\\&=\frac{a\psi _c''\overline{\hat{z}}^2}{2+2am}+ \left( \hat{z}{\mathcal {K}}_{k-1}{\hat{g}}_{k-1}+\overline{\hat{z}}{\mathcal {K}}_{k+1}{\hat{g}}_{k+1} \right) +{\mathcal {G}}_k \end{aligned} \end{aligned}$$

and for $$k\in \{-3,-4, \ldots \}$$, $$\langle \nu _k^*,P_sB_1(\hat{x}+\hat{y},\hat{x}+\hat{y})\rangle _{U}= \Big (\hat{z}{\mathcal {K}}_{k-1}{\hat{g}}_{k-1}+\overline{\hat{z}}{\mathcal {K}}_{k+1}{\hat{g}}_{k+1} \Big )+{\mathcal {G}}_k$$

Applying Ito’s formula on $d(\hat{g_k}\hat{g_l})$ for $k\in \{-3,-4 \ldots \}$ and $k+l=1$ we have:

$$\begin{aligned} \begin{aligned} d(\hat{g_k}\hat{g_l})&=\hat{g_k}d\hat{g_l}+\hat{g_l}d\hat{g_k}\\&=\varepsilon ^{-2}\lambda _l^s\hat{g_k}\hat{g_l}dt+ \varepsilon ^{-1} \left( \hat{z}{\mathcal {K}}_k{\hat{g}}_k{\hat{g}}_{-k}+\overline{\hat{z}}{\mathcal {K}}_{l+1}{\hat{g}}_k{\hat{g}}_{l+1} \right) dt+\varepsilon ^{-1}\hat{g}_k{\mathcal {G}}_ldt\\&\quad +\varepsilon ^{-2}\lambda _k^s\hat{g_k}\hat{g_l}dt+ \varepsilon ^{-1} \left( \hat{z}{\mathcal {K}}_l{\hat{g}}_l{\hat{g}}_{-l}+\overline{\hat{z}}{\mathcal {K}}_{k+1}{\hat{g}}_l{\hat{g}}_{k+1} \right) dt+\varepsilon ^{-1}\hat{g}_l{\mathcal {G}}_kdt \\&\quad +\varepsilon ^{-1}{\hat{g}}_k\sqrt{q_l} (d\beta _l(t)+id\beta _{-l}(t))+\varepsilon ^{-1}{\hat{g}}_l\sqrt{q_k} (d\beta _{-k}(t)-id\beta _{k}(t)); \end{aligned} \end{aligned}$$

for $k=-2$ and $l=3$ we have:

$$\begin{aligned} \begin{aligned} d(\hat{g_k}\hat{g_l})&=\hat{g_k}d\hat{g_l}+\hat{g_l}d\hat{g_k}\\&=\varepsilon ^{-2}\lambda _l^s\hat{g_k}\hat{g_l}dt+ \varepsilon ^{-1} \left( \hat{z}{\mathcal {K}}_k{\hat{g}}_k{\hat{g}}_{-k}+\overline{\hat{z}}{\mathcal {K}}_{l+1}{\hat{g}}_k{\hat{g}}_{l+1} \right) dt+\varepsilon ^{-1}\hat{g}_k{\mathcal {G}}_ldt+\varepsilon ^{-1}\hat{g}_l{\mathcal {G}}_kdt\\&\quad +\varepsilon ^{-2}\lambda _k^s\hat{g_k}\hat{g_l}dt+ \varepsilon ^{-1} \left( \hat{z}{\mathcal {K}}_l{\hat{g}}_l{\hat{g}}_{-l}+\overline{\hat{z}}{\mathcal {K}}_{k+1}{\hat{g}}_l{\hat{g}}_{k+1} \right) dt+\varepsilon ^{-1} \left( \frac{a\psi _c''\overline{\hat{z}}^2g_3}{2+2am}\right) dt \\&\quad +\varepsilon ^{-1}{\hat{g}}_k\sqrt{q_l} (d\beta _l(t)+id\beta _{-l}(t))+\varepsilon ^{-1}{\hat{g}}_l\sqrt{q_k} (d\beta _{-k}(t)-id\beta _{k}(t)). \end{aligned} \end{aligned}$$

Therefore, for $k\in \{-3,-4 \ldots \}$ and $k+l=1$,

$$\begin{aligned} \begin{aligned} \hat{g_k}\hat{g_l}dt&=-\varepsilon \left( \frac{1}{\lambda _k^s+\lambda _l^s}\right) \left( \hat{z}{\mathcal {K}}_k{\hat{g}}_k{\hat{g}}_{-k}+\overline{\hat{z}}{\mathcal {K}}_{l+1}{\hat{g}}_k{\hat{g}}_{l+1}+\hat{g}_k{\mathcal {G}}_l \right) dt\\&\quad -\varepsilon \left( \frac{1}{\lambda _k^s+\lambda _l^s}\right) \left( \hat{z}{\mathcal {K}}_l{\hat{g}}_l{\hat{g}}_{-l}+\overline{\hat{z}}{\mathcal {K}}_{k+1}{\hat{g}}_l{\hat{g}}_{k+1}+\hat{g}_l{\mathcal {G}}_k \right) dt\\&\quad -\varepsilon \left[ \frac{{\hat{g}}_l\sqrt{q_k} (d\beta _{-k}(t)-id\beta _{k}(t))+{\hat{g}}_k\sqrt{q_l} (d\beta _{l}(t)+id\beta _{-l}(t))}{\lambda _k^s+\lambda _l^s}\right] ; \end{aligned} \end{aligned}$$

for $$k=-2$$ and $$l=3$$,

$$\begin{aligned} \begin{aligned} \hat{g_k}\hat{g_l}dt&=-\varepsilon \left( \frac{1}{\lambda _k^s+\lambda _l^s}\right) \left( \hat{z}{\mathcal {K}}_k{\hat{g}}_k{\hat{g}}_{-k}+\overline{\hat{z}}{\mathcal {K}}_{l+1}{\hat{g}}_k{\hat{g}}_{l+1}+\hat{g}_k{\mathcal {G}}_l \right) dt\\&\quad -\varepsilon \left( \frac{1}{\lambda _k^s+\lambda _l^s}\right) \left( \hat{z}{\mathcal {K}}_l{\hat{g}}_l{\hat{g}}_{-l}+\overline{\hat{z}}{\mathcal {K}}_{k+1}{\hat{g}}_l{\hat{g}}_{k+1}+\hat{g}_l{\mathcal {G}}_k \right) dt\\&\quad -\varepsilon \left( \frac{1}{\lambda _k^s+\lambda _l^s}\right) \left( \frac{a\psi _c''\overline{\hat{z}}^2g_3}{2+2am}\right) dt\\&\quad -\varepsilon \left[ \frac{{\hat{g}}_l\sqrt{q_k} (d\beta _{-k}(t)-id\beta _{k}(t))+{\hat{g}}_k\sqrt{q_l} (d\beta _{l}(t)+id\beta _{-l}(t))}{\lambda _k^s+\lambda _l^s}\right] , \end{aligned} \end{aligned}$$

and the result follows easily from a combination of the above. $\square$

## D Homogenization Results in Eq. (5.14)

D.1 $$\begin{aligned} N_1(\omega )= & {} - \frac{{\mathcal {K}}_1{\mathcal {K}}_2}{4\lambda _2^s} \left( 2\varPhi _\delta ^*g^*_2+\sum \limits _{k\in {\mathbb {Z}}\setminus \{0,\pm 1\}}^{k+l=2}g^*_kg^*_l\right) \end{aligned}$$

D.2 $$\begin{aligned} N_2(\omega )= & {} - \frac{{\mathcal {K}}_1}{4l_c}\left[ (l_{11}\psi _{c,\mu _c}'')\left( \varPhi _\delta ^{*2}+2\sum \limits _{k\in \{-2,-3, \ldots \}}g^*_kg^*_{-k}\right) -(l_{12}\mathcal {S}_{\mu _c}''')(\varPsi _\delta ^{*2}) \right] \end{aligned}$$

D.3 $$\begin{aligned} N_3(\omega )= & {} -\frac{{\mathcal {K}}_1{\mathcal {K}}_2}{4\lambda _2} g^*_3, \end{aligned}$$

D.4 $$\begin{aligned} N_4(\omega )= & {} -\frac{ {\mathcal {K}}_1^2}{4}g^*_{-2}\left( \frac{g^*_2}{\lambda _2^s}\right) - \frac{ {\mathcal {K}}_1^2}{4}(\varPhi _\delta ^*)(l_{11}\varPhi _\delta ^*+l_{12}\varPsi _\delta ^*) \end{aligned}$$

D.5 $$\begin{aligned} N_5(\omega )= & {} -\frac{ {\mathcal {K}}_1^2}{4}(\varPhi _\delta ^*)\left( \frac{g^*_2}{\lambda _2^s}\right) - \frac{ {\mathcal {K}}_1^2}{4}g^*_{2}(l_{11}\varPhi _\delta ^*+l_{12}\varPsi _\delta ^*) \end{aligned}$$

D.6 $$\begin{aligned} N_6(\omega )= & {} -\frac{{\mathcal {K}}_1^2}{4}\left( \sum _{k\in {\mathbb {Z}}\setminus \{0,\pm 1\}}^{k+l=1}g_k^*g_l^*\right) (l_{11}\varPhi _\delta ^*+l_{12}\varPsi _\delta ^*)-\frac{{\mathcal {K}}_1^2}{4}\left( \sum _{k\in {\mathbb {Z}}\setminus \{0,\pm 1\}}^{k+l=-1}g_k^*g_l^*\right) \left( \frac{g^*_2}{\lambda _2^s}\right) \nonumber \\ \end{aligned}$$

D.7 $$\begin{aligned} N_7(\omega )= & {} -{\mathcal {K}}_1\sum _{k\in \{-2,-3 \ldots \}}^{k+l=1}\left( \frac{ {\mathcal {K}}_kg^*_kg^*_{-k}+{\mathcal {K}}_lg^*_lg^*_{-l}}{\lambda _k^s+\lambda _l^s}\right) \end{aligned}$$

D.8 $$\begin{aligned} N_8(\omega )= & {} -{\mathcal {K}}_1\sum _{k\in \{-2,-3 \ldots \}}^{k+l=1}\frac{ {\mathcal {K}}_{l+1}g^*_kg^*_{l+1}+{\mathcal {K}}_{k+1}g^*_lg^*_{k+1}}{\lambda _k^s+\lambda _l^s} \end{aligned}$$

D.9 $$\begin{aligned} N_9(\omega )= & {} -{\mathcal {K}}_1\sum _{k\in \{-2,-3 \ldots \}}^{k+l=1}\left( \frac{ g^*_k{\mathcal {G}}_l+g^*_l{\mathcal {G}}_k}{\lambda _k^s+\lambda _l^s}\right) \end{aligned}$$

D.10 $$\begin{aligned} N_{10}(\omega )= & {} -\frac{{\mathcal {K}}_{1}{\mathcal {K}}_{2}g^*_3}{2(\lambda _{-2}^s+\lambda _3^s)} \end{aligned}$$

D.11 $$\begin{aligned} N_{11}(\omega )= & {} -\frac{{\mathcal {K}}_1\psi _c'''}{2\psi _{c,\mu _c}''}g_3^*\end{aligned}$$

D.12 $$\begin{aligned} N_{12}(\omega )= & {} -\frac{{\mathcal {K}}_1\psi _c'''}{2\psi _{c,\mu _c}''}\left[ \varPhi _\delta ^{*2}+2\sum \limits _{k\in \{-2,-3 \ldots \}} g_k^*g_{-k}^*\right] \end{aligned}$$

D.13 $$\begin{aligned} N_{13}(\omega )= & {} -\frac{{\mathcal {K}}_1\psi _c'''}{2\psi _{c,\mu _c}''}\left[ 2\varPhi _\delta ^{*}g_2^*+\sum \limits _{k\in {\mathbb {Z}}\setminus \{0,\pm 1\}}^{k+l=2} g_k^*g_{l}^*\right] \end{aligned}$$

D.14 $$\begin{aligned} N_{14}(\omega )= & {} -\frac{{\mathcal {K}}_1\psi _c'''}{6\psi _{c,\mu _c}''}\left[ 3\varPhi _\delta ^{*}\left( \sum \limits _{k\in {\mathbb {Z}}\setminus \{0,\pm 1\}}^{k+l=1}g_k^*g_l^*\right) +\sum \limits _{k,l\in {\mathbb {Z}}\setminus \{0,\pm 1\}}^{k+l+m=1}g_k^*g_l^*g_m^*\right] \end{aligned}$$

## Footnotes

1 Note that $\hat{S}(t)$ should be dependent on $\hat{\mathfrak {q}}$. Since we investigate the solution for a fixed $\hat{\mathfrak {q}}$ at a time, we abuse the notation and do not emphasize the dependence on $\hat{\mathfrak {q}}$.

2 For $\hat{\mathfrak {q}}$ sufficiently close to 0, the second order expansion about $\mu _c$ can be omitted.

## References

[CR1] Arnold L, Sri Namachchivaya N, Schenk-Hoppé KR (1996). Toward an understanding of stochastic hopf bifurcation: a case study. Int. J. Bifurc. Chaos.

[CR2] Bahouri H, Chemin JY, Danchin R (2011). Fourier Analysis and Nonlinear Partial Differential Equations.

[CR3] Ball, J.: Geometric Theory of Semilinear Parabolic Equations (lecture notes in mathematics, 840) (1982)

[CR4] Banaszuk A, Hauksson HA, Mezic I (1999). A backstepping controller for a nonlinear partial differential equation model of compression system instabilities. SIAM J. Control. Optim..

[CR5] Baxendale PH (1994). A stochastic hopf bifurcation. Probab. Theory Relat. Fields.

[CR6] Birnir B, Hou S, Wellander N (2007). Derivation of the viscous moore-greitzer equation for aeroengine flow. J. Math. Phys..

[CR7] Blömker D, Fu H (2020). The impact of multiplicative noise in spdes close to bifurcation via amplitude equations. Nonlinearity.

[CR8] Blömker D, Hairer M, Pavliotis G (2007). Multiscale analysis for stochastic partial differential equations with quadratic nonlinearities. Nonlinearity.

[CR9] Blömker D, Hongbo F (2020). The impact of multiplicative noise in SPDEs close to bifurcation via amplitude equations. Nonlinearity.

[CR10] Blömker D, Romito M (2015). Stochastic pdes and lack of regularity. Jahresber. Deutsch. Math.-Verein..

[CR11] Da Prato G, Zabczyk J (2014). Stochastic Equations in Infinite Dimensions.

[CR12] Ethier SN, Kurtz TG (2009). Markov Processes: Characterization and Convergence.

[CR13] Gourdain N, Sicot F, Duchaine F, Gicquel L (2014). Large eddy simulation of flows in industrial compressors: a path from 2015 to 2035. Philos. Trans. R. Soc. A Math. Phys. Eng. Sci..

[CR14] Gravdahl JT (1998). Modeling and control of surge and rotating stall in compressors. J. Turbomach..

[CR15] Greitzer, E., Moore, F.: A theory of post-stall transients in axial compression systems: part ii-application (1986)

[CR16] Guckenheimer J, Holmes P (2013). Nonlinear Oscillations, Dynamical Systems, and Bifurcations of Vector Fields.

[CR17] Hairer, M.: An introduction to stochastic pdes. arXiv preprint arXiv:0907.4178 (2009)

[CR18] Hou S (2002). Solutions of Multidimensional Hyperbolic Systems of Conservation Laws by Discontinuous Galerkin Methods and a Derivation of the Moore-Greitzer Equation Using Homogenization.

[CR19] Kim T, Abed EH (1999). Closed-loop stability monitoring of axial flow compression systems. Nonlinear Dyn..

[CR20] Lord GJ, Powell CE, Shardlow T (2014). An Introduction to Computational Stochastic PDEs.

[CR21] Mohammed WW, Blömker D, Klepel K (2014). Multi-scale analysis of spdes with degenerate additive noise. J. Evol. Equ..

[CR22] Moore, F.K., Greitzer, E.M.: A theory of post-stall transients in axial compression systems: Part i-development of equations (1986)

[CR23] Pazy A (2012). Semigroups of Linear Operators and Applications to Partial Differential Equations.

[CR24] Stroock DW, Varadhan SS (2007). Multidimensional Diffusion Processes.

[CR25] Sviridenko M (1990). Martingale approach to limit theorems for semi-markov processes. Theory Probab. Appl..

[CR26] Xiao M (2008). Quantitative characteristic of rotating stall and surge for moore-greitzer pde model of an axial flow compressor. SIAM J. Appl. Dyn. Syst..

[CR27] Xiao M, Basar T (2000). Center manifold of the viscous moore-greitzer pde model. SIAM J. Appl. Math..

